# Protein kinases PknA and PknB independently and coordinately regulate essential *Mycobacterium tuberculosis* physiologies and antimicrobial susceptibility

**DOI:** 10.1371/journal.ppat.1008452

**Published:** 2020-04-07

**Authors:** Jumei Zeng, John Platig, Tan-Yun Cheng, Saima Ahmed, Yara Skaf, Lakshmi-Prasad Potluri, Daniel Schwartz, Hanno Steen, D. Branch Moody, Robert N. Husson

**Affiliations:** 1 Division of Infectious Diseases, Boston Children’s Hospital, Harvard Medical School, Boston, MA, United States of America; 2 Channing Division of Network Medicine, Brigham and Women’s Hospital, Harvard Medical School, Boston, MA; 3 Division of Rheumatology, Immunity and Inflammation, Brigham & Women’s Hospital, Harvard Medical School, Boston MA, United States of America; 4 Department of Pathology, Boston Children’s Hospital, Harvard Medical School, Boston, MA, United States of America; 5 Department of Physiology and Neurobiology, University of Connecticut, Storrs, CT, United States of America; National Institutes of Health, UNITED STATES

## Abstract

The *Mycobacterium tuberculosis* Ser/Thr protein kinases PknA and PknB are essential for growth and have been proposed as possible drug targets. We used a titratable conditional depletion system to investigate the functions of these kinases. Depletion of PknA or PknB or both kinases resulted in growth arrest, shortening of cells, and time-dependent loss of acid-fast staining with a concomitant decrease in mycolate synthesis and accumulation of trehalose monomycolate. Depletion of PknA and/or PknB resulted in markedly increased susceptibility to β-lactam antibiotics, and to the key tuberculosis drug rifampin. Phosphoproteomic analysis showed extensive changes in protein phosphorylation in response to PknA depletion and comparatively fewer changes with PknB depletion. These results identify candidate substrates of each kinase and suggest specific and coordinate roles for PknA and PknB in regulating multiple essential physiologies. These findings support these kinases as targets for new antituberculosis drugs and provide a valuable resource for targeted investigation of mechanisms by which protein phosphorylation regulates pathways required for growth and virulence in *M*. *tuberculosis*.

## Author summary

Tuberculosis is a major global health threat, with approximately 10 million new cases per year and 1.5 million deaths. Although tuberculosis is treatable, cure requires prolonged antibiotic courses and strains that are resistant to current treatments are increasingly common. Understanding how *Mycobacterium tuberculosis* adapts to the human host during infection can lead to new approaches to treat tuberculosis. A major adaptive mechanism is reversible protein phosphorylation mediated by protein kinases, which detect signals in the extracellular environment and transmit this information to regulate *M*. *tuberculosis* physiology. In this study we analyzed two *M*. *tuberculosis* kinases, PknA and PknB, by comparing strains in which these kinases are present to strains in which one or both of these kinases has been depleted. We identified features that differ between the kinase replete and kinase depleted strains including bacterial growth and viability, cell shape, the bacterial cell envelope and gene regulation. We also identified proteins that show decreased phosphorylation in the kinase depleted strains, which can result from direct or indirect effects of kinase depletion. These findings provide insight into how these kinases are important for *M*. *tuberculosis* pathogenesis and indicate that these kinases may be valuable targets for development of new TB drugs.

## Introduction

The World Health Organization estimates that in 2018 10 million people developed tuberculosis (TB) and 1.5 million died from the disease [1]. Although tuberculosis is curable with antibiotics, current lengthy treatment regimens make completion of therapy difficult. Moreover, incomplete adherence to these prolonged treatment courses favors the emergence of rifampin-resistant/multidrug-resistant TB. Treatment of these resistant strains requires even longer courses with more drugs that are often poorly tolerated, resulting in lower adherence and rates of cure [2].

In addition to TB disease, *Mycobacterium tuberculosis* can cause an asymptomatic latent infection that can last for many years [3]. *M*. *tuberculosis* encounters many distinct environments in the host during the course of asymptomatic infection and active disease. These include short and long-term intracellular niches within phagocytes, and extracellular sites in a broad range of tissues and in necrotic caseum. *M*. *tuberculosis* requires mechanisms to sense signals in these host environments to adaptively regulate its cell physiology. Bacterial signal transduction mechanisms are also required for manipulation of host defenses by *M*. *tuberculosis*, a hallmark of tuberculosis pathogenesis. Together, these responses to host environments allow bacterial persistence and replication during *M*. *tuberculosis* infection and disease.

Reversible protein phosphorylation is a broadly conserved mechanism of transmembrane signaling that regulates cell physiology in response to signals from the extracellular environment. In most well-studied bacteria, two component systems are the predominant form of phosphorylation-based signal transduction [4]. In other bacteria, including *M*. *tuberculosis* and other Actinobacteria, Ser/Thr and Tyr protein kinases also play a major role in transmembrane signal transduction [5–10]. Ser/Thr and Tyr phosphorylation is the dominant phosphorylation-based signal transduction mechanism in eukaryotes, and aberrant function of kinase pathways has been linked to human disease, including several forms of cancer. As a result, several Tyr and Ser/Thr kinases have been targeted by small molecule inhibitors, many of which are in clinical use as approved drugs [11]. The conserved domain structure and mechanism of action of eukaryotic and bacterial protein kinases provide a foundation for developing small molecule inhibitors of essential bacterial Ser/Thr kinases that could serve as leads for drug development [12]. The success of such an approach would depend on optimizing selectivity for bacterial versus human kinases; that over 50 drugs approved for clinical use selectively target specific eukaryotic kinases [11], suggests that the development of potent and selective inhibitors of *M*. *tuberculosis* essential kinases may be achievable.

*M*. *tuberculosis* has 11 Ser/Thr kinases, two of which PknA and PknB (which also phosphorylates Tyr), are essential for bacteria viability [13, 14]. In prior studies, we and others have investigated the function of these kinases using genetic and chemical biology approaches [6, 8, 10, 15–21]. These investigations provide evidence that these kinases regulate, via reversible phosphorylation of regulators and enzymes, many functions required for *M*. *tuberculosis* growth and viability, including peptidoglycan synthesis and turnover, cell division, lipid metabolism, translation and central carbon metabolism [10, 17, 19, 22–24]. Consistent with their being essential for in vitro growth, depletion of PknA or PknB has been shown to result in lack of replication and markedly decreased lung pathology in a mouse model of infection [15, 16].

Knowledge of the direct substrates of the *M*. *tuberculosis* Ser/Thr kinases is essential for understanding their function and several protein substrates have been proposed for PknA and/or PknB, supported by evidence of varying strength [17]. Despite extensive study, the overlapping and discrete regulatory roles of *M*. *tuberculosis* PknA and PknB are not well understood.

Here we used a genetic depletion system in *M*. *tuberculosis* to investigate these kinases individually and as a pair. We compared kinase replete versus kinase-depleted bacteria using a combination of microscopy and microbiological, biochemical and phosphoproteomic approaches. We identified effects of kinase depletion on growth, viability, cell morphology, acid-fast staining and antimicrobial susceptibility. These phenotypes, together with concomitant changes in mycolic acid metabolism and extensive differences in protein phosphorylation between kinase replete and depleted strains, identify multiple components of the mycobacterial cell envelope, as well as several other essential cell physiologies, as targets of phosphorylation-based regulation by *M*. *tuberculosis* PknA and PknB. Our findings indicate distinct but partially overlapping roles for these essential *M*. *tuberculosis* kinases, support their potential as targets for anti-tuberculosis drug development, and provide a new resource for investigation of mechanisms by which reversible protein phosphorylation regulates pathways that are essential for growth and virulence of *M*. *tuberculosis*.

## Results

### Conditional expression of *pknA*, *pknB and pknA*+*pknB*

**Fig 1:**
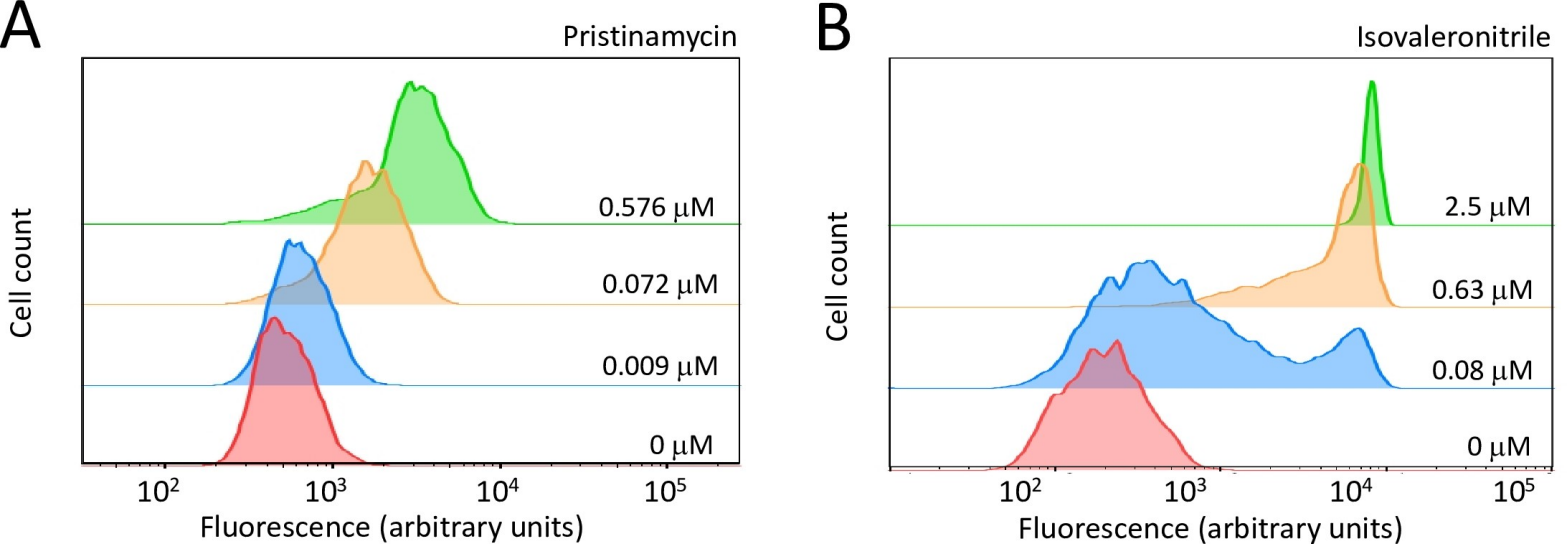
FACS analysis of titration of pptr-regulated gene expression. **1A)** Titration of pristinamycin induction of mCherry expression under control of the pptr promoter was analyzed in an attenuated strain of *M*. *tuberculosis* (Δ*leuD*, Δ*panCD* [55]). Cells were induced for 48 hours, followed by fluorescence intensity measurement by FACS analysis to show the distribution of fluorescence intensity of individual cells. **1B)** The same reporter under control of the nitrile inducible promoter, which is not titratable at the single cell level [27], was used as a control. Data are representative of biological triplicates.

The genes encoding PknA and PknB are co-expressed in an operon in the *M*. *tuberculosis* chromosome. We used a pristinamycin (ptc)-inducible expression system [25] to conditionally deplete both *pknA* and *pknB* together or *pknB* individually in *M*. *tuberculosis* (**S1A Fig**). To achieve depletion of *pknA* alone, we complemented the *pknA*+*pknB* depletion strain with a *pknB* allele regulated by an anhydrotetracycline (atc)-inducible promoter (**S1A and S1B Fig**). Though the ptc-inducible promoter has been used for regulated gene expression in mycobacteria [25], it has not been shown to be titratable at the single cell level. Because this can be critical in interpreting phenotypes, we measured fluorescence intensity of individual cells containing an mCherry reporter [26] expressed from the ptc-inducible promoter. We found that this promoter is titratable at the single cell level, with graded increased activity in individual cells in response to increased inducer concentrations, shown by increased fluorescence Intensity (**Fig 1**). In comparison, a nitrile-inducible promoter that we previously characterized [27] is titratable primarily at the population level, manifested by a greater proportion of cells with increased fluorescence of similar intensity in response to increased nitrile concentration.

**Fig 2:**
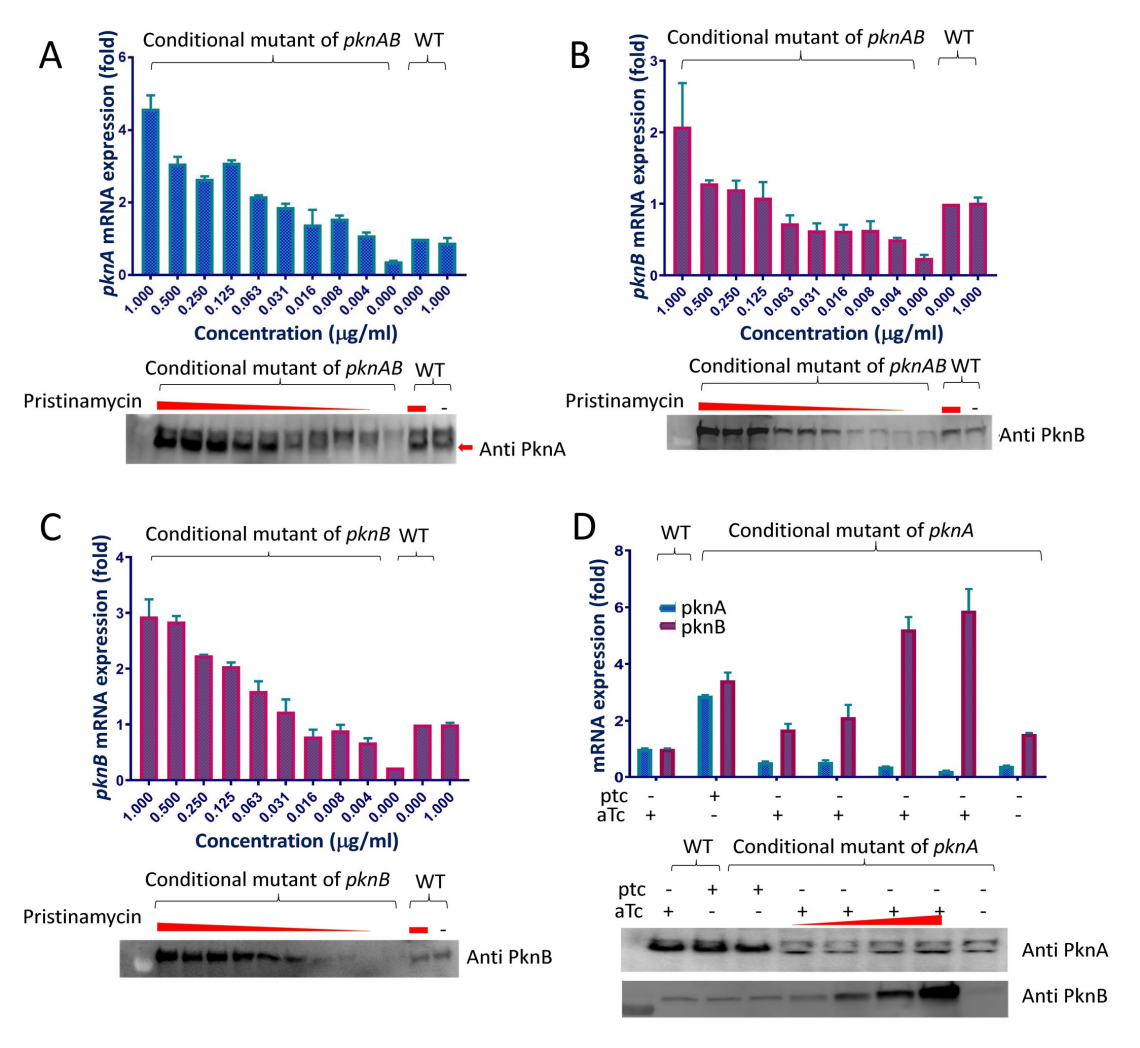
Titration of kinase expression in the depletion strains. Cells were grown in the presence of the indicated concentrations of ptc or atc for four days, followed by total RNA and protein isolation as described in the methods. For each panel the graph shows mRNA relative quantification by RT-qPCR and the image below each graph shows protein relative quantification by Western blotting using polyclonal rabbit anti-PknA antibody or monoclonal mouse anti-PknB antibody. Equal amounts of total RNA were used in each lane for RT-qPCR and equal amounts of total proein were loaded into each well for Western blotting. **2A)** titration of *pknA* mRNA and PknA protein in the *pknA+pknB* depletion strain in response to 2-fold dilutions of ptc. **2B)** titration of *pknB* mRNA and PknB protein in the *pknA+pknB* depletion strain. The membrane from panel 2A was stripped and re-probed with anti-PknB antibody. **2C)** titration of *pknB* mRNA and PknB protein in the *pknB* depletion strain. **2D)** titration of *pknA* and *pknB* mRNA and PknA and PknB protein in the *pknA* depletion strain. For these experiments, concentration of ptc when present was 0.25 μg/ml and concentrations of anhydrotetracycline were 10, 20, 40, or 200 ng/mL. Note that that there is a non-specific band that is present immediately above the PknA-specific band in panels 2A and 2D.

Because both kinase overexpression and depletion can affect growth, we titrated the expression of *pknA* and *pknB* to identify ptc concentrations that achieve kinase mRNA and protein levels similar to those present in wild type *M*. *tuberculosis* (**Fig 2A–2C**). To be able to analyze a strain in which only *pknA* was depleted, we determined the concentration of atc that achieved *pknB* expression in the *pknA*+*pknB* depletion strain that is similar to *pknB* expression in wild type (**Fig 2D**).

### Growth and cell morphology effects of kinase depletion

**Fig 3:**
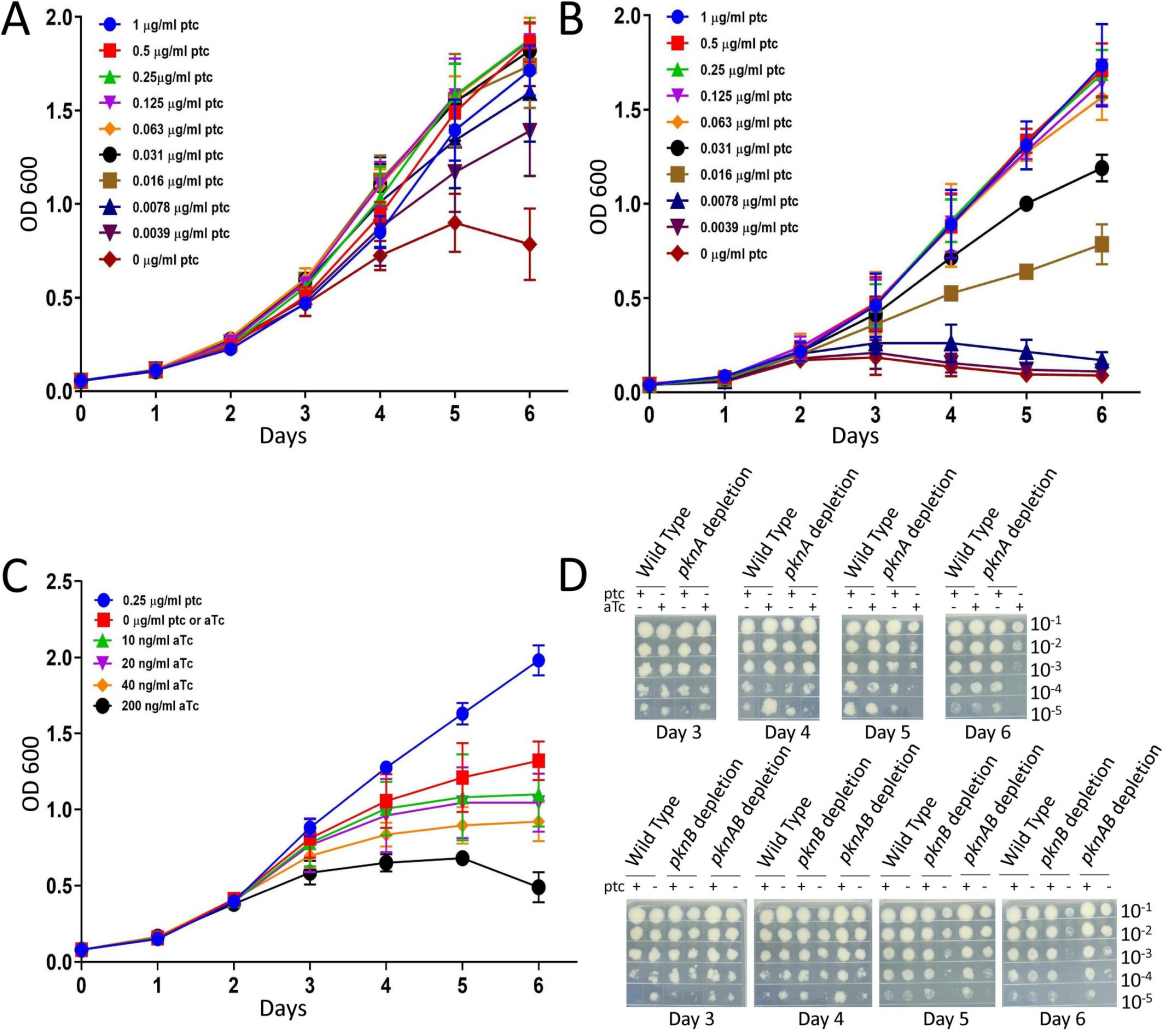
Growth of kinase depletion strains. Cultures were pre-grown in 7H9-ADN-Tw with 0.25 μg/ml ptc, pelleted, washed and diluted to achieve an initial OD_600_ of 0.01. **3A)** growth curve of *pknA+pknB* depletion mutant with titration of ptc induction. **3B)** growth curve of *pknB* depletion mutant with titration of ptc induction, **3C)** growth curve of *pknA* depletion mutant with 0.25 μg/ml ptc or without induction, plus titrated induction of *pknB* by atc. Data for 3a-3c are the average of three biological replicates and error bars represent +/- 1 SD. **3D)** All three mutants were grown in liquid culture with or without 0.25μg/ml ptc induction; the atc concentration for the *pknA* depletion mutant was 20ng/ml. Serial dilutions (10^−1^ to 10^−5^) of all three mutants were spotted on 7h9 agar at day 3, day 4, day 5 and day 6. Data are representative of duplicate experiments.

All three depletion strains failed to form colonies when plated onto agar lacking inducer, confirming that *pknA* and *pknB* are both individually essential for growth (**S1C Fig**) [15]. We also performed growth curves in liquid medium over a range of inducer concentrations. Consistent with previous results [15, 16], we observed time-dependent growth defects in liquid medium for each strain in the absence of inducer (**Fig 3A–3C**). Depletion of *pknA* and especially *pknB* individually had more severe growth phenotypes than did depletion of both *pknA* and *pknB* together. We also observed decreases in cell viability after 4 days of kinase depletion, with *pknA* and *pknB* single depletion strains both showing greater decreases in colony forming units over time compared to *pknA*+*pknB* depletion (**Figs 3D and S2**). The more severe phenotypes of the *pknA* and *pknB* single depletion strains strain suggest that absence of one kinase may result in dysregulation of the activity of the non-depleted kinase in a manner that interferes with growth. A previous report showed a more severe growth phenotype for a *pknA+pknB* depletion strain generated using the same single crossover approach that we have used. The reason for this difference is not known, but may reflect differences in growth conditions, inducer concentrations or treatment of the cultures prior to initiation of depletion.

**Fig 4:**
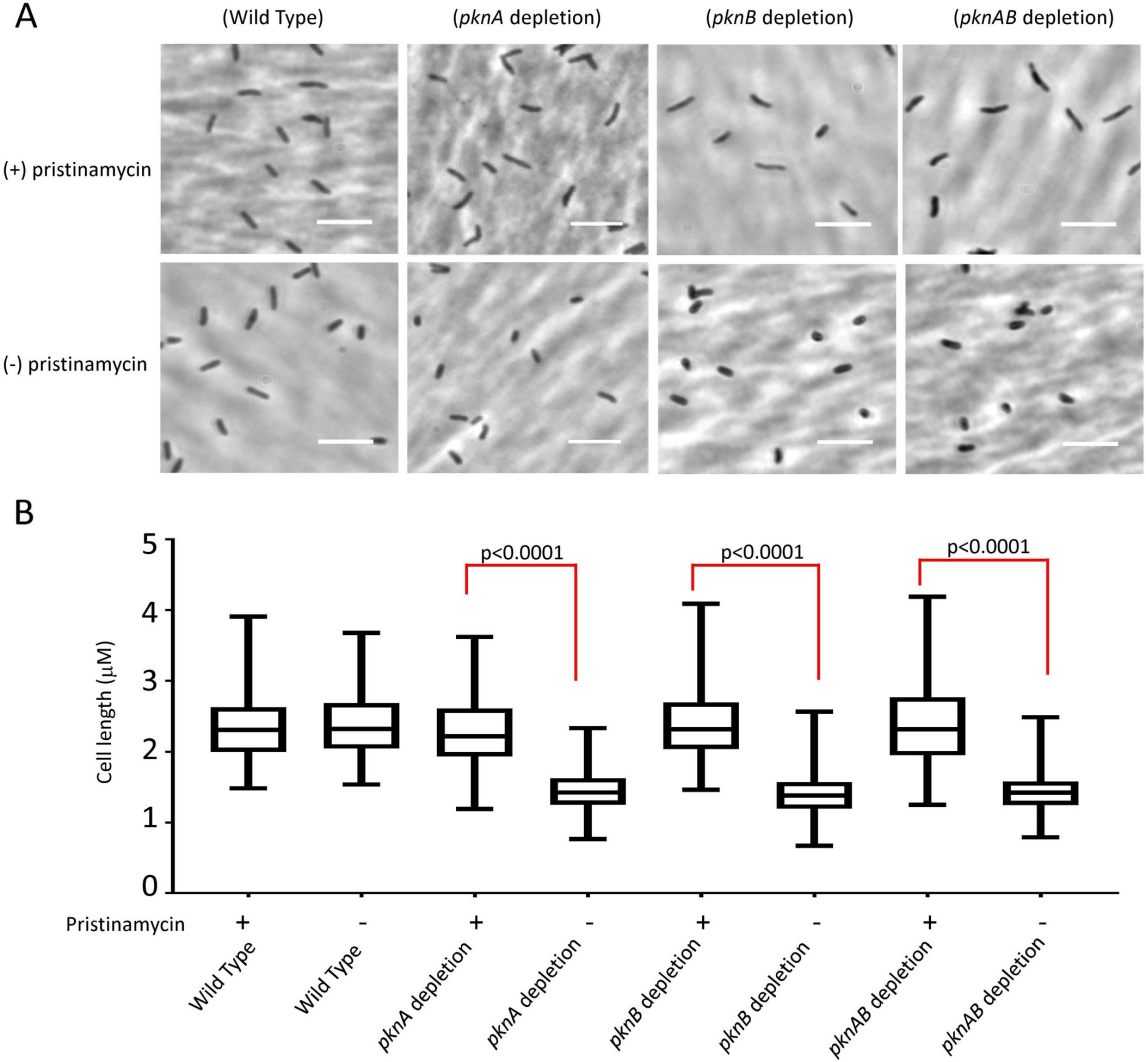
Morphology of wild type *M*. *tuberculosis* cells and cells from the *pknA*, *pknB* or *pknA*+*pknB* depletion strains. **4A)** Phase contrast microscopy of cells from wild type, *pknB*, or *pknA*+*pknB* depletion strain in the presence (0.25 μg/ml) or absence of ptc. For *pknA* depletion cells were grown in 0.25 μg/ml of ptc or 20 ng/ml atc. Pictures were taken with a 100x objective. Size bar = 5μM. **4B)** Distribution of length measurements of cells of wild type, *pknA*, *pknB* and *pknA*+*pknB* depletion mutants obtained with ImageJ software. More than 200 cells were counted for each strain. The boxes represent the middle 50% of measurements and the whiskers the minimum to maximum values of the measurements.

The growth defects seen in the kinase depletion strains led us to examine the morphology of *M*. *tuberculosis* cells following kinase depletion. Both single depletion strains and the *pknA*+*pknB* depletion strain showed significant cell shortening in the absence of kinase induction (**Fig 4A and 4B**). Given the localization of both kinases to the cell poles and septum, this phenotype may result from dysregulated cell division and/or polar growth [6, 10, 21]. A potential contributor the growth and morphology effects is the decreased phosphorylation in the PknB depletion strain of MmpS3 (LamA), recently described as a divisome protein that inhibits growth at the new cell pole following cell division [28]. The maintenance of rod-shaped morphology of these cells contrasts with the unipolar ballooning of cells in which *divIVA* (*wag31*), which is required for peptidoglycan synthesis at sub-polar sites, is depleted [29, 30].

### Loss of acid-fast staining and altered mycolic acid metabolism in response to *pknA* and *pknB* depletion

**Fig 5:**
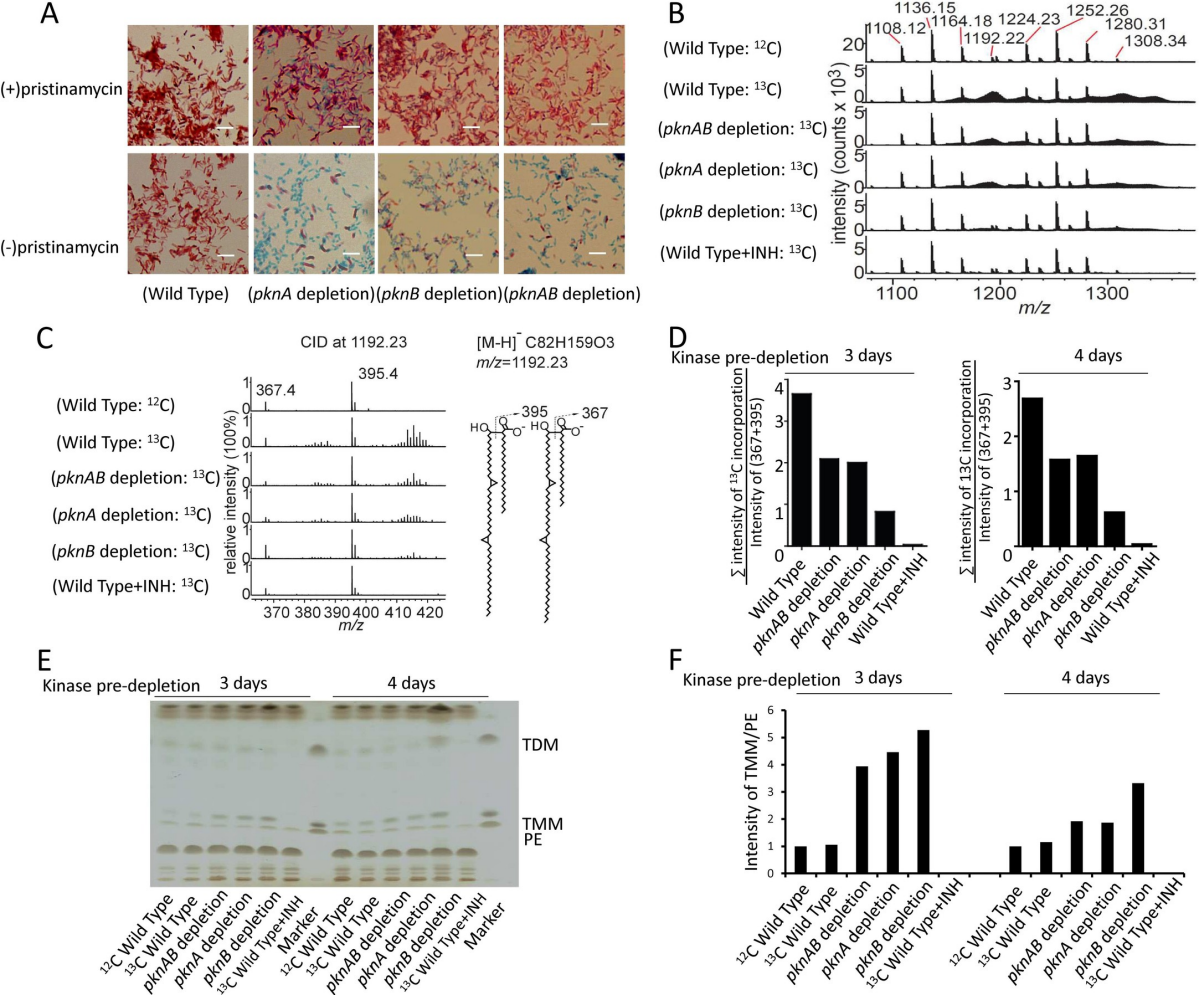
Acid-fast staining and mycolic acid synthesis in *pknA*, *pknB* and *pknA+pknB* depletion mutants. **5A)** Acid-fast staining of *M*. *tuberculosis* wild type, *pknA*, *pknB* and *pknA+pknB* depletion strains was carried out with or without ptc induction for 6 days. Pictures were taken with a 100X objective on a Nikon Eclipse TE2000-E inverted microscope. Size bar = 5μM. **5B)** Detection of newly synthesized mycolic acids by ^13^C labeling. *M*. *tuberculosis* wild type and kinase depletion strains were grown for 48h in liquid culture medium with or without 3 mg/mL of [1, 2-^13^C] acetate prior to harvesting cells. The total pool of cell-wall-bound mycolic acids was isolated by saponification and analyzed by high performance liquid chromatography-mass spectrometry (HPLC-MS) in the negative-ion mode. Mass spectra of mycolic acids shown here are representative of biological duplicates. **5C)** The m/z 1192.23 ion and all of the ions with ±2.0 m/z intervals around this precursor ion were subject to collision induced mass spectrometry (CID-MS) and generation of C24 or C26 fatty acid fragments. The unlabeled mycolic acid (^12^C82H159O3, *m/z* 1192.23) produced only m/z 367.358 (^12^C24H47O2) and 395.389 (^12^C26H51O2), whereas other isotopically enriched mycolic acids produced two extra clusters of higher mass value ^13^C-enriched fatty acids. The right panel shows the two predicted ions corresponding to the CID fragments. Data are representative of duplicates. **5D)** Newly synthesized MAs were assessed by the ratio of the total intensity of ^13^C incorporation peaks to the intensity of unlabeled fatty acids (m/z 367 and 395) at 5 and 6 days after the onset of depletion (kinase pre-depletion 3 and 4 days, then 2 days with [1, 2-^13^C] acetate treatment). The CID-MS data of m/z 1192 was used for this analysis. ^13^C labeling was assessed based on the extent of new isotope detection from the CID-MS simplified background values. **5E)** Thin layer chromatography of lipids and glycolipids of *M*. *tuberculosis* from wild type and kinase depletion strains at 5 and 6 days after the onset of depletion (kinase pre-depletion 3 and 4 days, then 2 days with [1, 2-^13^C] acetate treatment). Standards for TDM, TMM and PE were run to the right of the experimental samples at each time point. **5F)** Quantification by ImageJ software of TMM at 5 and 6 days after the onset of depletion (kinase pre-depletion 3 and 4 days, then 2 days with [1, 2-^13^C] acetate treatment), normalized to phosphatidylethanolamine (PE) as the loading control. Panels 5D,E,F show representative data from 2 different depletion samples.

Based on prior work with a PknA and PknB inhibitor showing broad effects on the mycobacterial cell envelope [6, 19], we investigated whether kinase depletion affected acid fast staining, which results from dye-retention in the abundant mycolic acids and glycolipids in the mycobacterial cell envelope [31]. We observed a marked decrease in acid-fast staining of all 3 strains, first evident at day 4 following onset of kinase depletion, with most cells becoming non-acid-fast by day 6 (**Fig 5A**). This finding is consistent with progressive alteration of the cell envelope in the absence of PknA and PknB activity.

We therefore investigated whether this phenotype was associated with changes in total or newly synthesized mycolic acids in the kinase replete versus depleted strains. TLC of total mycolates in the depleted strains compared to induced strains did not show changes when normalized to whole cell lipids (**S3A Fig**). We then used a newly developed method to examine de novo synthesis of mycolates by mass spectrometry of cells grown in the presence of ^13^C acetate [32] (**Fig 5B**). This assay works on the principle that collision induced dissociation mass spectrometry allows reliable isolation of a precursor molecule in a narrow mass window, which largely removes signals from naturally occurring isotopes, allowing new isotopes from ^13^C labeling to be reliably identified. These experiments showed clear and reproducible decreases in new synthesis of mycolates in the kinase depleted versus kinase replete strains (**Fig 5C**).

This approach was used to further investigate the degree of isotope labeling of the affected lipids (**Fig 5D**). We observed consistent decreases in all three depletion strains, though less severe than observed with isoniazid treatment, which completely eliminates mycolic acid synthesis. As a specificity control, we analyzed C26 free fatty acids (m/z 395.39) in the total lipid extracts to evaluate ^13^C incorporation. We observed isotope enrichment for the wild type treated by INH and in all depletion strains compared to the unlabeled wild type (**S3B Fig**). This result indicates that all strains are able to take up ^13^C acetate, so that the observed differences between the wild type and the depletion strains were specifically due to altered de novo mycolate biosynthesis.

**Fig 6:**
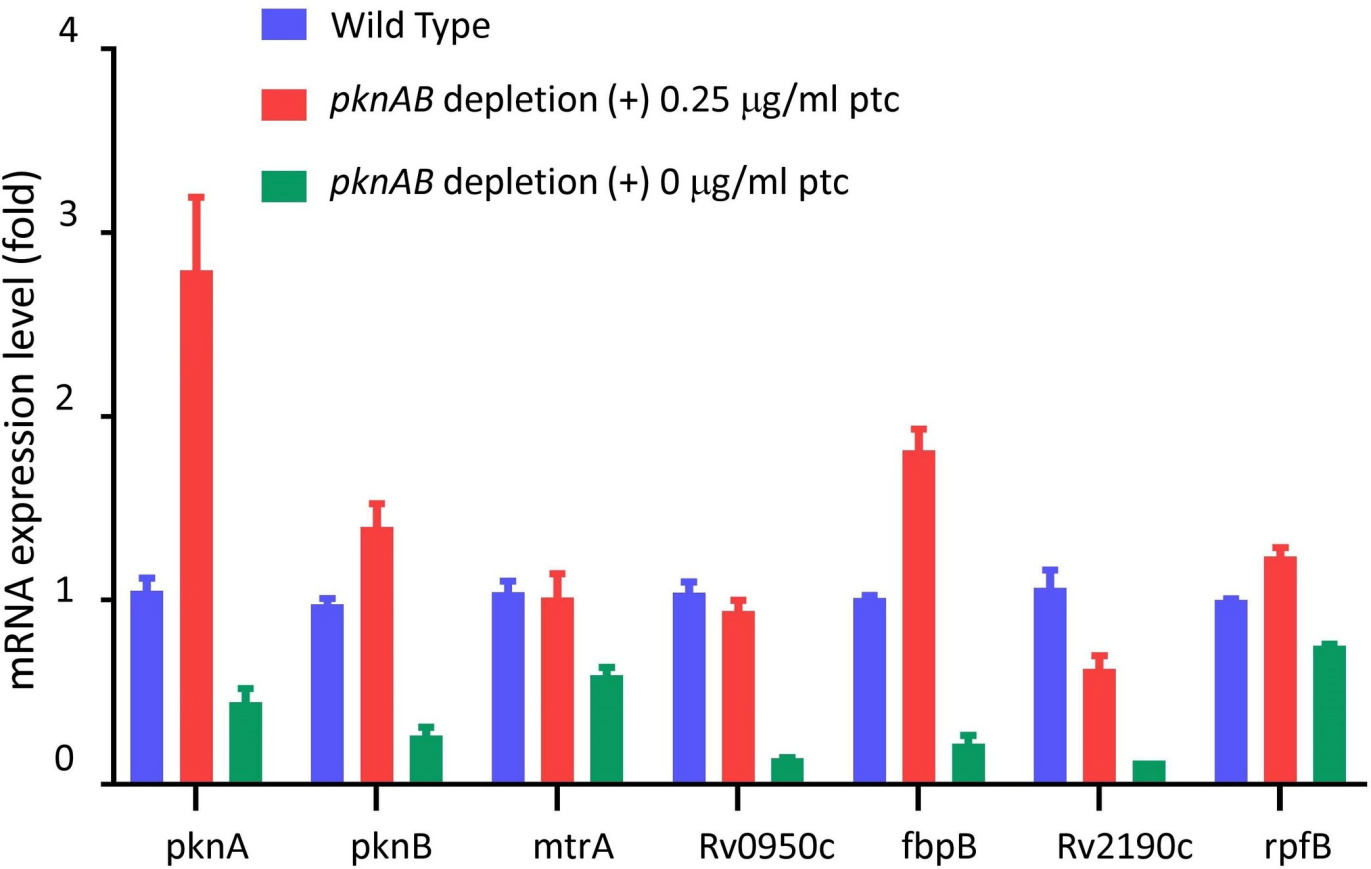
Relative quantification by RT-qPCR of MtrA-regulated gene expression. The *pknA*+*pknB* depletion strain was grown with or without 0.25 μg/ml ptc and wild type *M*. *tuberculosis* was grown without ptc. Cells were harvested at day 4 and RNAs were extracted with Trizol as previous described [56]. RT-qPCR was performed and the data were analyzed using the ΔΔCT method. Biological duplicate experiments were performed with technical replicates in each experiment. Error bars show +/- 1 S.D.

Finally, we examined the abundant mycolyl-glycolipids trehalose monomycolate (TMM) and trehalose dimycolate (TDM) by TLC. In replicate experiments we saw increases of TMM in all three depletion strains, though we did not see consistent changes in TDM (**Fig 5E and 5F**). The accumulation of TMM in the depletion strains suggests decreased conjugation of TMM to produce TDM by the action of mycolyl transferases FbpA and FbpB. We previously demonstrated that expression of *fbpB* is repressed by binding of the response regulator MtrA to the *fbpB* promoter-region and that phosphorylation of Thr213 in MtrA disrupts this binding [6], leading us to propose a mechanism linking decreased MtrA phosphorylation with decreased FbpB-mediated conjugation resulting in accumulation of TMM. Consistent with this mechanism we found decreased expression of *fpbB* (4.6-fold vs. wild type) and other MtrA-regulated genes in the *pknA+pknB* depletion strain (**Fig 6)**, where we observed significantly decreased phosphorylation of MtrA both at the previously identified Thr213 residue, and at Thr217, a new phosphorylation site identified in this study. In addition, we found that phosphorylation of Mmpl3, the “flippase” of TMM across the inner mycobacterial membrane [33], is significantly decreased at 2 sites in the *pknA* depletion strain, which may also affect the levels of TMM and the rate of conjugation to TDM.

### Increased antibiotic susceptibility in the setting of *pknA* and *pknB* depletion

Deletion or inhibition of PknB orthologues in Gram-positive bacteria can lead to increased susceptibility to β-lactam antibiotics [34–36], and meropenem has been shown to potentiate the activity of a PknB inhibitor 2-fold in auxotrophic *M*. *tuberculosis* [37]. Though carbapenems have been used to treat MDR-TB, cephalosporins and penicillins are generally not active against *M*. *tuberculosis* at clinically relevant concentrations. We therefore investigated whether representative β-lactams would have increased activity against the kinase-depleted strains. Under conditions of partial kinase depletion that allowed sufficient growth to test for antibiotic activity, we found that the carbapenems meropenem and faropenem, and the cephalosporins cefotaxime and ceftriaxone were markedly potentiated (2 to 32-fold) for inhibiting growth in the setting of *pknA* or *pknB* depletion, with lesser potentiation (1 to 8-fold) when both *pknA* and *pknB* were depleted (**Table 1)**. We did not observe increased susceptibility to isoniazid, ethambutol and levofloxacin. Rifampin, however, showed 2-8-fold potentiation in the depletion strains. The differences in potentiation of the different antibiotic classes likely reflect their distinct mechanisms of action, uptake, egress and/or metabolism that are differently affected by altered activity of PknA or PknB. These findings also suggest that an inhibitor of PknB or PknA could be useful in TB treatment regimens both as a direct acting TB drug and as a potentiator of the activity of β-lactam antibiotics and rifampin.

**Table 1:** Lowest inhibitory concentrations of antibiotics in wild type and kinase depletion strains (μg/ml).

	Faropenem	Cefotaxime	Ceftriaxone	Meropenem	Rifampin	Ethambutol	Isoniazid	Levofloxacin
Wild Type	2–4	4–8	8	1–4	0.0125	1.25	0.16	0.31
*pknA* depletion	0.25	0.25–0.5	0.25–0.5	0.25–0.5	0.0031–0.0063	ND	0.078	ND
*pknB* depletion	0.25	2	0.25	0.125	0.0015	0.625	0.078	0.15
*pknAB* depletion	2	4	4	0.5	0.0015	0.625	0.078	0.15

ND: Not done.

Because of the up to two-fold overexpression of *pknB* in the *pknA* depletion strain when *pknB* is induced with 20 ng/ml of atc, we performed MABA assays for cefotaxime and meropenem to compare the effects of induction with 10 vs. 20 ng/ml of atc. We observed the same effect of *pknA* depletion on susceptibility to these two β-lactam antibiotics at both atc concentrations (**S4 Fig**). We also examined the acid-fast staining phenotype of this strain and found similar effects on this phenotype when the strain was grown in the presence of 10 or 20 ng/ml of atc (**S5 Fig**). These two results, together with the growth curve data showing nearly identical growth of the *pknA* depletion strain with either 10 or 20 ng/ml of atc to induce *pknB* expression (**Fig 3C)**, indicate that these phenotypes result primarily from effects of *pknA* depletion rather than from differences in *pknB* expression in this strain.

### Differential phosphorylation in kinase depletion strains and candidate substrate identification

We compared phosphorylation in each of the kinase depletion strains grown without inducer, to the same strains in which kinase expression was induced. We obtained samples on day 4 for *pknA* and *pknB* single depletion, and day 5 for *pknA*+*pknB* double depletion, based on experiments showing the onset of a clear growth defect but limited cell death in the depleted strains at these time points (**Figs S6 and 3D**). Because depletion occurs over days, differences in protein phosphorylation will reflect both direct effects of PknA and/or PknB depletion and downstream effects that result from changes in the activity of other kinases, as well as possible differences in the rate of depletion of the two kinases or in rates of turnover of individual phosphoproteins. For the *pknA* depletion only, the up to 2-fold overexpression of *pknB* may have contributed to the differential phosphorylation results observed with this strain.

Overall, we observed Ser, Thr or Tyr phosphorylation of 712 proteins for which one or more phosphorylation sites were present in at least 8 of 18 samples analyzed in at least 1 strain (**S2 Table**). Using criteria of log_2_FC <-1 and adjusted P <0.05, we identified 302 unique sites in 204 proteins that showed significantly decreased phosphorylation in one or more kinase-depleted strain compared to the same strain with induced kinase expression. We also identified 299 unique phosphorylation sites in 201 proteins that were significantly increased (log_2_FC >1, adjusted P <0.05) (**S2 Table**). While most proteins were phosphorylated on one to three sites, some were phosphorylated on many sites, the most striking examples being FhaA and MmpS3, which are phosphorylated on 23 and 16 unique residues, respectively.

**Fig 7:**
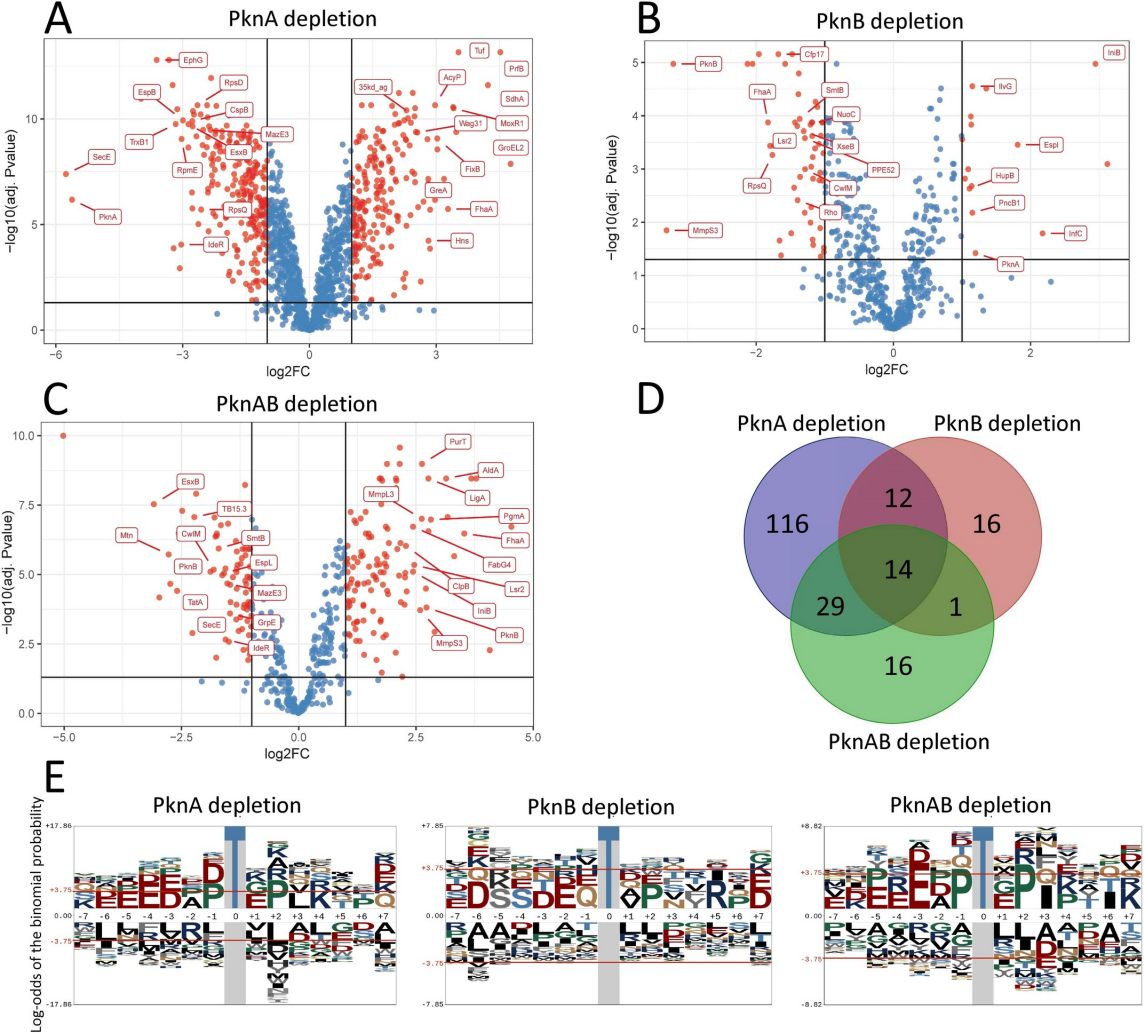
Phosphoproteomic analysis of kinase depletion strains. **7A, 7B, 7C)** Volcano plots of phosphopeptides in the indicated kinase depletion strains shown as the ratio (Log_2_FC) of peptide abundance in the kinase depleted vs. kinase replete strains, as described in the Methods. Significantly differentially abundant phosphopeptides (Log2FC <-1.0 or >1.0 and adj. P<0.05) are shown in red. Data are from 3 biological replicates with 3 separate cultures in each replicate for each strain. **7D)** Venn diagram showing overlap between strains of the proteins from which significantly decreased phosphopeptides in the kinase depleted compared to the kinase replete samples were observed. **7E)** pLogos showing relative statistical significance of amino acids at positions adjacent to the phosphoacceptor Thr in phosphopeptides that were significantly decreased in each kinase depletion strain. The red horizontal line indicates P = 0.05 after Bonferroni correction. Additional pLogos for significantly decreased Ser-centered and for increased Thr and Ser-centered phosphopepetides are in S9 Fig.

A surprising finding was the large number of phosphorylation sites that showed decreased phosphorylation in the *pknA* depletion strain, where 234 unique phosphorylation sites in 171 proteins were significantly decreased (**Fig 7A and S3 Table**). In the *pknB* depletion strain, phosphorylation was significantly decreased at 52 unique sites in 43 proteins, and in the *pknA*+*pknB* depletion strain at 74 unique sites in 60 proteins (**Fig 7B and 7C; S3 Table)**. Confirming specific kinase depletion, phosphorylation was significantly decreased at 6 sites in PknA and none in PknB in the *pknA* depletion strain, and phosphorylation of 3 sites in PknB and none in PknA were decreased in the *pknB* depletion strain. In the *pknA* + *pknB* depletion strain, two sites in PknA and one site in PknB were significantly decreased.

Identification of direct substrates of a kinase can provide insight into mechanisms by which protein phosphorylation by that kinase alters cell physiology. The patterns of decreased phosphorylation of specific peptides in the depletion strains allow identification of candidate substrates of PknA and PknB. We identified candidate PknA substrates using criteria of i) significantly decreased phosphorylation with *pknA* depletion and ii) increased or not significantly changed phosphorylation with *pknB* depletion. Similarly, significantly decreased phosphorylation with *pknB* depletion and increased or not significantly changed phosphorylation with *pknA* depletion identified candidate PknB substrate sequences. Sites with significantly decreased phosphorylation in both the *pknA* and *pknB* depletion strains were identified as candidate substrates of both PknA and PknB. Using these criteria we identified 127 candidate PknA substrate sequences, 28 candidate PknB substrate sequences, and 15 candidate PknA + PknB substrate sequences (**S4 Table**). At the protein level we identified 104 candidate PknA substrates, 23 candidate PknB substrates and 14 candidate PknA + PknB substrates. Given the non-comprehensive nature of phosphoproteomics, and its inability to distinguish direct from indirect effects of kinase depletion, these candidate substrate identifications will need to be tested using targeted approaches. As described below, however, these results clearly identify several pathways required for growth and virulence that are affected by depletion of PknA and/or PknB.

To examine the extent of overlap of the proteins and phosphopeptides with decreased phosphorylation among the three depletion strains we performed Venn diagram and correlation analyses. Of 43 proteins with decreased phosphorylation sites in the *pknB* depletion strain, 26 (60%) also had decreased sites in the *pknA* and 15 (35%) in the *pknA+pknB* depletion strains (**Fig 7D**). Of 60 proteins with decreased phosphorylation in the *pknA+pknB* depletion strain, 43 (72%) were decreased in the *pknA* strain including 14 common to all three strains. At the peptide level, we saw less overlap between the *pknA* and *pknB* depletion strains, where 14 of 52 (27%) unique phosphopeptides with decreased phosphorylation in the *pknB* depletion strain were also decreased in the *pknA* strain (**S7A and S7B Fig**). Similarly, among all quantified phosphopeptides, we observed no correlation between abundance of individual phosphopeptides with *pknA* vs. *pknB* depletion, or with *pknB* depletion vs. *pknA*+*pknB* depletion. However, there was moderate correlation (r = 0.438) between the *pknA* and *pknA*+*pknB* depletion strains (**S7C–S7E Fig**). These findings suggest PknA has a very broad role in regulating *M*. *tuberculosis* via Ser/Thr phosphorylation, and that while PknA and PknB regulate many of the same proteins and pathways, the manner in which the do so often differs. They also suggest that when both kinases are depleted, differences in Ser/Thr phosphorylation predominantly reflect the loss of PknA activity.

We also compared the phosphoproteins showing decreased phosphorylation in the kinase depletion strains to phosphoproteins that were decreased in a recent study of *pknB* depletion, as well as to our prior results identifying proteins with decreased phosphorylation in response to a chemical inhibitor of both PknA and PknB [6, 24]. In each case we observed partial overlap between the results from this study and the prior findings (**S8A–S8C Fig**). We also compared the total quantified phosphoproteins identified in this study to those found in the kinase inhibitor study and to an earlier phosphoproteomic study in which we identified phosphorylation sites in *M*. *tuberculosis* at different growth stages and in response to stresses [7]. We found that 69% of all phosphoproteins in the kinase inhibitor study and 43% of all phosphoproteins in stress phosphoproteome study were present in the quantified phosphoproteins identified in the current study (**S8D Fig**).

We searched the differentially phosphorylated peptides for conserved sequence motifs using the pLogo algorithm [38] and identified distinct patterns in the amino acid sequences surrounding the phosphoacceptor Thr for phosphopeptides with decreased or increased phosphorylation in the *pknA*, *pknB* and *pknA*+*pknB* depletion strains (**Figs 7E and S9).** The pLogo for phosphopeptides with significantly decreased Thr phosphorylation in the *pknA* depletion strain showed significant enrichment of acidic residues at -1, -3 through -6, and at +7; Pro at -1 and +2; hydrophobic residues at +3 (Leu, Val); and basic residues at +4 (Lys, Arg). In the *pknB* depletion strain we noted enrichment of specific residues at several positions, though none reached statistical significance, likely because of the small number of phosphopeptides analyzed. The motif from the *pknA*+*pknB* strain was highly similar to the *pknA* depletion strain motif. The *pknA* and *pknB* depletion strain motifs were less similar, consistent with the limited overlap in phosphopeptides with decreased phosphorylation between these strains (**S7A Fig**). These motifs also showed similarity to those we identified in phosphopeptides with decreased phosphorylation in the kinase inhibitor experiments, most notably the acidic residues at -3 and Pro at +2 [6].

### PknA and PknB target pathways required for growth and virulence

Several processes that are essential for growth were identified in the differential phosphorylation data. Consistent with prior findings, these data include phosphorylation-mediated regulation of peptidoglycan synthesis by PknA and PknB. In the *pknA* depletion strain we identified significantly decreased phosphorylation of sites in the essential peptidoglycan synthesis regulators Wag31 (DivIVA) and CwlM (**S3 Table**) [21, 29]. In the *pknB* depletion data we identified a Thr phosphorylation site with significantly decreased phosphorylation in CwlM and 3 sites with decreased phosphorylation in FhaA, an essential forkhead-associated domain protein that interacts with CwlM and MviN, the essential peptidoglycan flippase. Strikingly, FhaA (15 sites including all 3 sites with decreased phosphorylation in the *pknB* strain), Wag31 (4 sites), and MviN (2 sites) showed increased phosphorylation in the *pknA* depletion strains. These findings indicate that PknA and PknB target essential peptidoglycan synthesis regulators to control *M*. *tuberculosis* growth, but in distinct ways.

The quantitative phosphoproteomic data also show protein secretion to be a major target of PknA and PknB-mediated phosphorylation. In the *pknA* depletion strain we identified decreased phosphorylation of three sites in SecE, an essential protein of the general secretion system and 1 site in FtsY, the signal particle receptor of this system. Two of the sites in SecE also showed decreased phosphorylation in the *pknB* and *pknA+pknB* depletion strains and a distinct site in FtsY also showed decreased phosphorylation in the *pknA+pknB* depletion strain. Additionally, four sites in TatA, an essential channel forming protein of the twin-arginine transport system showed decreased phosphorylation in the *pknA* strain, one of which was also decreased in the *pknA+pknB* depletion strain, and a distinct TatA site had decreased phosphorylation in the *pknB* depletion strain. Further, five proteins in the virulence-associated ESX-1 secretion system showed decreased phosphorylation in the *pknA* depletion strain: one site each in the ESX-1 regulator/nucleoid associated protein EspR, the immunodominant secreted antigen EspF, and the ESX-1-associated protein EspL, as well as four and six unique sites, respectively, in the secreted virulence proteins EspB and EsxB. Three EsxB sites also had decreased phosphorylation in the *pknA+pknB* depletion strain as did the single EspL site and a distinct EspF site. In the *pknB* depletion strain, only a unique EsxB site showed decreased phosphorylation. These findings suggest that the three major protein secretion systems of *M*. *tuberculosis* are regulated by PknA and/or PknB-mediated protein phosphorylation.

Translation also appears to be extensively regulated by PknA and PknB. In the *pknA* depletion strain, nineteen ribosomal proteins, the initiation factors InfA and InfC, and the peptide chain release factor-2 PrfB showed decreased phosphorylation. In contrast, initiation factor Tuf and two distinct PrfB phosphopeptides were three of the five phosphopeptides with the greatest increase in phosphorylation in this strain. In the *pknB* depletion strain, only three ribosomal proteins showed decreased phosphorylation, suggesting a more limited role for PknB in translation control.

Several proteins involved in transcription regulation were significantly differentially phosphorylated in the kinase depletion strains. In the *pknA* depletion strain, in addition to specific transcription regulators including SmtB, IdeR, RegS3, and PhoP, proteins expected to have broad effects on transcription showed decreased phosphorylation, including the transcript cleavage factor GreA, the primary sigma factor SigA, and the nucleoid-associated proteins HupB (HU) and EspR. In the *pknB* depletion strain two different proteins with broad effects on transcription, the nucleoid-associated protein Lsr2 and the termination protein Rho, showed significantly decreased phosphorylation. These data point to both targeted and broad regulation of transcription by PknA and PknB, but with distinct roles for each kinase.

Our data also indicate kinase-mediated regulation of cell division and DNA synthesis, key aspects of the bacterial cell cycle. Phosphorylation of FtsQ, an essential protein involved in septum formation, was significantly decreased in the *pknA* depletion strain but increased in the *pknA*+*pknB* depletion strain; several other cell division proteins (FtsB, FtsE, FtsK and FtsZ) were phosphorylated in one or more strains but did not meet our significance criteria. Essential DNA synthesis proteins were also affected, with the single strand DNA binding protein Ssb showing significantly decreased phosphorylation in the *pknA* depletion strain and the replication initiator protein DnaA showing significantly increased phosphorylation. DnaN, the DNA polymerase III initiator, showed increased phosphorylation just below our change threshold. Significantly decreased phosphorylation of the response regulator MtrA was seen in the *pknA*+*pknB* depletion strain. We recently showed phosphorylation-regulated binding of cognate promoter region DNA by MtrA [6], a response regulator that has been implicated in regulating initiation of chromosome replication, peptidoglycan turnover and glycolipid synthesis. Consistent with the prior finding of increased MtrA binding and repression of MtrA-regulated gene expression in the absence of MtrA phosphorylation, we observed decreased expression of MtrA-regulated genes in the *pknA*+*pknB* depletion strain, where MtrA shows decreased phosphorylation (**Fig 6**) [39–41].

Each of the above pathways was targeted in our kinase inhibitor data [6], though in most cases less extensively and often at different sites compared to these kinase depletion data. We also identified new processes affected by PknA and PknB-mediated phosphorylation. A notable example is decreased phosphorylation of several antitoxin proteins of the VapBC and MazEF toxin-antitoxin modules in the *pknA* depletion data. This result suggests that PknA-mediated signal transduction is a regulatory mechanism for the action of the toxin proteins, which typically degrade specific RNAs resulting in growth arrest, antibiotic and other stress tolerance, and remodeling the *M*. *tuberculosis* proteome [42–44].

In summary, these quantitative phosphorylation data identify numerous proteins of core cell physiologies required for growth and virulence as targets of phosphorylation by PknA and PknB (**S3 Table, Fig 7**). Taken together, these data indicate substantial overlap in pathways targeted by these kinases, but with distinct activities of PknA and PknB.

## Discussion

Reversible Ser/Thr and Tyr phosphorylation is increasingly recognized as an important component of transmembrane signal transduction in bacteria. PknB orthologues in Gram-positive bacteria have been shown to regulate peptidoglycan metabolism, morphogenesis and susceptibility to cell wall active antibiotics [34, 35, 45, 46]. In mycobacteria and other actinobacteria, the presence of several transmembrane Ser/Thr kinases indicates an extensive role for protein phosphorylation in regulating cell processes in response to extracytoplasmic signals. In *M*. *tuberculosis*, an obligate human pathogen, these kinases must sense and transduce host signals to allow the pathogen to adapt, survive and cause disease.

In previous work we identified proteins in a number of pathways that were differentially phosphorylated in response to treatment with a small molecule inhibitor of both PknA and PknB [6]. In this study, using the titratable ptc-regulated promoter system, we examined the effects of *pknA* and *pknB* depletion, individually and in combination, allowing us to focus on the effects of depleting each kinase while avoiding potential off-target effects that can occur with chemical inhibition.

An unexpected result from this work is the greater number of proteins that showed decreased phosphorylation in the *pknA* depletion strain compared to the *pknB* depletion strain and the relatively limited overlap in differential phosphorylation between these strains. This finding clearly indicates a major role for PknA in regulating the *M*. *tuberculosis* phosphoproteome, but it is possible that limitations of our approach may have affected these results. For example, although we titrated RNA and protein for each kinase at day 4 following onset of depletion, we do not know whether PknA and PknB have different rates of decay of over time. If PknA decays more quickly, for example this might lead to relative underestimation of the number of proteins targeted by PknB compared to PknA. Further, distinct decay rates for individual phosphoproteins may have affected our detection of significantly decreased phosphorylation of some proteins in our experiments. Finally, our use of an atc concentration that results in up to two-fold higher than wild type levels of *pknB* expression in the *pknA* depletion strain may have affected the differential phosphorylation that we detected in this strain.

The manner in which PknA and PknB interact functionally is not well understood. Early work showed that PknA and PknB can cross-phosphorylate each other [8], though later data suggested unidirectional trans-phosphorylation of PknA by PknB, leading to the hypothesis that PknB regulates PknA activity [47]. Subsequent work, however, showed that PknA autophosphorylates in the mycobacterial cell and that PknA abundance is unaltered when PknB is depleted [15, 48]. Our data showing limited overlap in peptides that are differentially phosphorylated in the *pknA* versus *pknB* depletion strains argue against a pathway in which PknB activates PknA. Rather, our data provide evidence of coordinate regulation of several pathways but with distinct regulatory roles for PknA and PknB, via phosphorylation of different sites in the same protein, or of different proteins within the same or related pathways.

We identified altered phosphorylation in the depletion strains of proteins in several pathways required for growth, suggesting that these kinases broadly adapt *M*. *tuberculosis* physiology to regulate growth, as has been shown for PknB in the setting of hypoxia-mediated growth arrest and renewed growth with reaeration [49]. Consistent with our kinase inhibitor data [6], the kinase depletion results also indicate regulation by PknA and PknB of the mycobacterial cell envelope. Decreased phosphorylation of sites in FhaA, and CwlM in the *pknB* depletion strain support the previously identified role for PknB in regulating peptidoglycan synthesis [6, 10, 21, 24, 50]. In the *pknA* depletion strain, the differential phosphorylation of the peptidoglycan synthesis regulators Wag31, CwlM and FhaA, and of the MviN peptidoglycan flippase, newly identify a major but distinct role for PknA in regulating peptidoglycan synthesis.

The time-dependent loss of acid-fast staining in the setting of kinase depletion suggests alterations in the lipid content and/or structure of the mycobacterial envelope, particularly the outer membrane. We observed decreased de novo mycolic acid synthesis in the kinase depletion strains and accumulation of TMM, consistent with decreased mycolate conjugation to produce TDM. Though levels of TDM did not clearly change in our TLC analysis, our prior kinase inhibitor study showed small decreases in TDM following kinase inhibition and demonstrated decreased abundance of the mycolyl transferase protein FbpB (antigen 85b) [6]. Here we determined that *fbpB* expression, which is repressed by binding of unphosphorylated MtrA, is markedly reduced in the *pknA*+*pknB* depletion strain. Together, these results provide a candidate mechanism for our findings, i.e. that decreased FbpB activity in the setting of kinase depletion contributes to accumulation of TMM, despite decreased de novo mycolate synthesis. Changes in mycolate conjugation also suggest a possible mechanism for decreased acid-fast staining. However, the stable levels of total saponifiable mycolates suggest that the location and membrane configuration of mycolyl lipids are relevant, and that additional mechanisms may play a role, potentially including phosphorylation-dependent changes in MmpL3 flippase activity or changes in the structure of other components of the cell envelope that alter dye retention.

Differential phosphorylation of transmembrane protein secretion pathways, including the general secretory and twin arginine transport pathways, as well as Type VII secretion systems, particularly the virulence-associated ESX-1 pathway, further highlights targeting of the pathogen-host interface by PknA and PknB. Individually depleting *pknA* or *pknB*, for example, led to decreased phosphorylation of the same two sites in SecE, a component of the general secretory translocon, indicating that both kinases regulate this secretion system. In contrast, four sites in TatA showed decreased phosphorylation only in the *pknA* depletion stain. In the ESX-1 system *pknA* depletion led to decreased phosphorylation of the EspR regulatory protein, the ESX-1 associated protein EspF, whereas the secreted substrate protein EsxB (Cfp10) showed decreased phosphorylation, though at distinct sites, in the *pknA* and the *pknB* depletion strains.

In addition to cell envelope structure and functions, we identified several other core pathways that are essential for growth and viability that are differentially phosphorylated in the kinase depletion strains. These include translation, where 18 and 3 ribosomal proteins showed decreased phosphorylation with *pknA* and *pknB* depletion, respectively. Several transcription proteins also showed decreased phosphorylation in the *pknA* strain and *pknB* depletion strains, including transcription factors that specifically regulate small sets of genes, as well as proteins that have broad, genome scale activities. These include the primary sigma factor SigA, the nucleoid-associated proteins MihF and HupB, and the transcription elongation factor GrpE in the *pknA* depletion strain, and the nucleoid-associated protein Lsr2 and the transcription termination protein Rho in the *pknB* depletion strain. These findings suggest broad targeting of transcription by both kinases, but via differential phosphorylation of distinct proteins.

The differential phosphorylation of key regulators of peptidoglycan synthesis and the changes in mycolate synthesis and conjugation in the kinase depletion strains provide candidate mechanisms for the increased antibiotic susceptibility that we observed. The marked increased susceptibility to β-lactams of both single depletion strains, and to a lesser extent the *pknA* + *pknB* strain, mirrors similar observations with deletion of *pknB* orthologues in Gram-positive bacteria where this gene is not essential [34]. Though the specific changes in peptidoglycan resulting from kinase depletion or inhibition have not been determined, the data from this study, together with our prior transcriptomic and metabolomic data showing a block in diaminopimelate synthesis in the setting of kinase inhibition [6], suggest effects on peptidoglycan precursor availability that could lead to incomplete or abnormal peptidoglycan structure and increased susceptibility to β-lactam antibiotics. The effects of *pknA* and *pknB* depletion and inhibition on mycolate metabolism suggest a mechanism by which alterations in the lipid and glycolipid components of the cell envelope could enhance entry of the highly lipophilic drug rifampin to achieve increased concentrations and activity in the *M*. *tuberculosis* cell. We identified one site (Thr764) in the beta subunit of RNA polymerase, the protein in which nearly all rifampin resistance conferring mutations occur, that showed increased phosphorylation in the *pknA* depletion strain. Though this site is not in the rifampin resistance determining region where most resistance mutations occur [51], it is possible that phosphorylation of this site might affect rifamycin susceptibility.

The magnitude of decreases in lowest inhibitory concentrations of the β-lactams in the kinase-depleted strains suggests that kinase inhibitor-β-lactam combinations have the potential to be clinically useful additions to anti-tuberculosis therapy, both for their direct effects and for their potentiation of β-lactam activity. Current research trials are testing increased rifampin doses to accelerate *M*. *tuberculosis* killing and shorten tuberculosis treatment. The magnitude of the potentiation of rifampin activity in all three depletion strains suggests that kinase inhibitor-rifamycin combination could be a valuable component of anti-tuberculosis therapy by enhancing rifamycin activity *in vivo* without the increased toxicity associated with higher rifampin dosing.

In addition to identifying multiple essential pathways targeted by PknA and PknB, our findings lead to several questions regarding the role of these kinases in *M*. *tuberculosis* physiology. One area of particular interest is how PknA and PknB interact functionally. The phosphoproteomic data in this study suggest a broad role for PknA with a more limited, partially overlapping, but essential role for PknB, with both kinases targeting pathways required for growth and viability. That *pknA* and *pknB* are co-expressed from adjacent translationally coupled genes strongly suggests that these two kinases act in a coordinated manner. However, the limited overlap in differential phosphorylation and the presence of a transcription start site positioned to transcribe *pknB* alone [52], indicate that PknA and PknB likely act independently under some conditions. A second priority area, identification of direct *in vivo* substrates of PknA and PknB, will further inform the specific functions of each protein. Our differential phosphorylation data identifying candidate substrates for both kinases (**S4 Table**) provide a valuable resource for investigating how PknA and/or PknB-mediated phosphorylation affects protein function. These insights will be essential for understanding how these kinases contribute to tuberculosis pathogenesis and how they may be targeted to enhance anti-tuberculosis therapy.

## Materials and methods

### Strains, media, reagents, plasmids and primers

*M*. *tuberculosis* H37Rv was used as the wild type and as the parental strain for all mutants. *E*. *coli* TOP10 and XL1 blue were used for cloning and were grown in LB broth supplemented with appropriate antibiotics (25 μg/ml kanamycin or 150 μg/ml hygromycin). *M*. *tuberculosis* H37Rv was grown at 37°C in Middlebrook 7H9 liquid medium (Difco) supplemented with 0.5% albumin, 0.2% glucose, 0.085% NaCl, 0.2% Glycerol and 0.05% Tween 80 (7H9-ADN-Tw). Kanamycin (25 μg/ml) or hygromycin (50 μg/ml) was added to liquid or agar medium when appropriate. Restriction endonucleases and DNA modifying enzymes were purchased from New England Biolabs. Pristinamycin 1A (ptc) was purchased from Molcan Corporation (Canada). Anti-phosphothreonine antibody and Anti-rabbit IgG HRP-linked antibody were purchased from Cell Signaling Technology. Analytical grade chemicals and reagents were purchased from Sigma Aldrich. Details of primers, plasmids and strains are shown in S1 Table.

### Generation of conditional depletion strains in *M*. *tuberculosis*

Plasmid pMYT797 was kindly provided by Francesca Forti [25]. The *pknB* depletion strain was constructed through introducing pMYT797 into the *M*. *tuberculosis* chromosome by a single homologous recombination event as previous described [25] (**S1A Fig**). To generate the *pknA* + *pknB* conditional depletion strain, pMYT797 was digested by SphI, blunted, and subsequently NcoI digested. A 770 bp 5’ region of *pknA* (+1 to +770bp) was PCR-amplified using primers *JZ-1* and *JZ-2*. The PCR product was digested by NcoI and NruI, and ligated into pMYT797. The targeting construct thus obtained was electroporated into *M*. *tuberculosis* H37Rv. Candidate single cross-over transformants were obtained by hygromycin selection. PCR of genomic DNA from candidate strains, using primers annealing to flanking regions of *pknA-pknB*, yielded the expected 4kb PCR product, which was confirmed by sequencing. The Psmyc of pTE-28S15-0X was fused to *tetR* of pTE-10M-0X and cloned into PacI and ClaI sites of pTC-0X-1L [53]. To obtain the *pknA* depletion strain from the *pknA+pknB* depletion strain, *pknB* was PCR-amplified using primers JZ-5 and JZ-6 and cloned under control of the *uv15tetO* promoter in pTC-0X-1L-psmyc-tetR (pRH2522) [54] and transformed into the *pknA*+*pknB* depletion strain. Growth of this strain in the presence of anhydrotetracycline (atc) induces expression of *pknB*, resulting in the *pknA* depletion strain (**S1B Fig)**.

### Analysis of inducible gene expression by fluorescence-activated cell sorting (FACS) assays

To investigate whether the ptc inducible promoter (PIP) system can be titrated at the single cell level, we used FACS to compare mCherry expression regulated by the NitR-controlled promoter, to expression regulated by pptr. mCherry was cloned into the pNit vector and pRH2046, and the resulting plasmids were electroporated into the *M*. *tuberculosis* auxotroph mc2-6206 (H37Rv ΔleuD ΔpanCD::hyg) [26, 55]. The transformants were selected by kanamycin for pNit-mCherry and spectinomycin for pRH2046-mCherry. Individual colonies were inoculated into 7H9+ADN+leucine+pantothenate and grown for 3 weeks. The bacteria were diluted to an optical density at 600nm of 0.2 and ptc was added at 0.576 μM, 0.072 μM, 0.009 μM, 0 μM, or isovaleronitrile was added at 2.5 μM, 0.63 μM, 0.08 μM, 0 μM. Cells were harvested after inducing 48 h, resuspended in PBS and filtered through a 35-um cell strainer (Cat# 352235, BD Falcon). Single cell fluorescence was determined using a MACSQUANT-VYB flow cytometer (Miltenyi Biotec, Bergisch Gladbach, Germany). Analyses were carried out using FlowJo Software.

### Assessing gene essentiality for replication and viability

To characterize the growth of the depletion mutants, the strains were grown to logarithmic phase (OD_600_ 0.8–1.0) in the presence of ptc. Cells were collected, washed, and then diluted to OD_600_ of 0.03–0.05 in 7H9-ADN-Tw with or without ptc. Two-fold dilutions of ptc were added from 1 μg/ml to 0.0039 μg/ml, cultures were incubated at 37°C with shaking and the OD_600_ was recorded daily. Wild type H37Rv was cultured in parallel. Experiments were performed in triplicate, and the average OD_600_ values were plotted as a function of time. Cultures were also grown with and without ptc induction, washed and then plated at serial time points on Middlebrook 7H9 agar with ptc induction to assess for viability of each depletion strain over time (**S1C Fig**).

### RNA and total protein extraction

For analysis of *pknA* and *pknB* expression in the depletion mutants, strains were grown with different ptc concentrations as described above. Samples from *M*. *tuberculosis* cultures were taken at day 4 and day 6 for isolation of RNA and total protein. Cells were harvested and treated as previously described [56]. RNA was extracted, purified and treated with Dnase I and Turbo DNAse (Thermo Fisher). Quantity and quality of RNA was determined using a Nanodrop instrument (Thermo Fisher). Proteins were extracted and solubilized in 9.5 M urea/2% CHAPS, pH 9.1.

### RT-qPCR

Reverse transcription (RT) and quantitative PCR of cDNA (RT-qPCR) were performed on equal amounts of total RNA for each condition, using the Quanta Biosciences qScript cDNA synthesis kit and the PerfeCTa-SYBR green Supermix, respectively as previously described [56]. Data were analyzed using the ΔΔCT method with *M*. *tuberculosis sigA* as the reference gene [57]. Biological duplicate samples were analyzed for each target sequence.

### Western blotting

For western blotting, 10 μg of total protein for each condition was resolved by SDS-PAGE followed by transfer to a PVDF membrane, which was probed with primary rabbit polyclonal antibodies against PknA (1:3,000) or mouse monoclonal antibody against PknB (1:3000) [18] followed by HRP-linked anti-rabbit secondary antibody (1:10 000) (Cell Signaling Technology). The blot was incubated with LumiGLO chemiluminescent substrate (Cell Signaling Technology) and detected using a Kodak Image Station (Carestream Health).

### Acid-fast staining

Strains were grown in 7H9-ADN-Tw medium, pelleted and fixed in 4% paraformaldehyde in PBS for 2 h. Fixed cells were pelleted, washed and resuspended in PBS. 10 μl of cell suspension were spread onto a glass slide. The slides were heated, stained with the TB Carbolfuchsin KF kit (Becton Dickinson) following the manufacturer’s instructions. Slides were rinsed with distilled water, dried, and 10 μl of Cytoseal 60 was placed on top of the sample and covered with a coverslip. A Nikon Eclipse 55i microscope was used to observe acid fast stained cells.

### Microscopy

For examination of cell morphology, cells were grown, harvested, fixed and resuspended in PBS as described above. 10 μl of the cell suspension was spotted on a 1% agarose coated glass slide to immobilize the cells. A coverslip was placed on the slide and cell morphology was visualized using a Nikon Eclipse TE2000-E inverted microscope fitted with a 100x Plan Achromatic phase contrast oil-immersion objective. Photographs were collected with a Hamamatsu ORCA-AG CCD camera, using IPLab imaging software. Measurement of cell length was performed using ImageJ software. Cell length in wild type, *pknA* depletion, *pknB* depletion and *pknA+pknB* depletion strains, with and without ptc induction, were compared using an unpaired t-test, using GraphPad Prism Software.

### Microplate Alamar Blue assay (MABA) for antibiotic susceptibility testing

*M*. *tuberculosis pknA*, *pknB*, *pknA+pknB* depletion strains were grown with ptc induction at 37°C in 7H9-ADC-Tw to OD_600_ ~0.8. The cells were pelleted, washed twice with 7H9-ADC-Tw and diluted to a calculated OD_600_ 0.001 in7H9-ADC-Tw. Each strain was placed in a 96-well clear bottom plate with 2-fold dilutions of ptc across the columns and 2-fold dilutions of antibiotic across the rows. Dilutions were made in 7H9-ADC-Tw and all wells contained 10 μg/ml clavulanic acid. Plates were incubated for 7–8 days and inhibitory concentrations of antibiotics were determined as previously described, based on color change following overnight incubation after addition of Alamar Blue, with pink indicating growth and blue indicating absence of growth. The lowest antibiotic concentration that results in absence of growth (blue well) correlates with minimal inhibitory concentrations obtained using traditional methods [58]. For the kinase depletion strains, we defined the lowest inhibitory concentration as the lowest antibiotic concentration that inhibited growth at the lowest ptc concentration that resulted in full growth in the absence of antibiotic. Biological duplicates were performed for each experiment.

### Protein sample preparation, mass spectrometry and phosphopeptide identification

Samples for phosphoproteomics were obtained from 3 independent experiments, in each of which 3 separate cultures with kinase depletion and 3 separate cultures without kinase depletion were grown and processed for phosphoproteomics as described below, resulting in 9 kinase-induced and 9 kinase depleted samples per strain that were treated as independent samples for calculation of differential abundance and P values. Protein samples were prepared as previously described [6, 59]. One mg protein samples were reduced with DTT and transferred into a 30 kDa MWCO ultrafiltration filter (Microcon-30 Centrifugal Filters, Millipore) for filter-aided sample processing [59], followed by washing with ABC solution (8 M Urea in 50 mM ammonium bicarbonate) and alkylated with 0.05 M iodoacetamide solution (IAA, TCI America). Trypsin digestion was carried out using a 1:50 enzyme to protein ratio with sequencing grade modified trypsin (Promega) by incubating at 37°C for 18 hours. The samples were desalted on an Oasis HLB column (1cc, Waters). The desalted samples were eluted with gradient acetonitrile: 500ul 30% ACN in 0.1% FA, then 300ul 50% ACN in 0.1% FA, at last 300ul 70% ACN in 0.1% FA. Samples were ready for phosphopeptide enrichment after evaporating to dryness overnight (Vacufuge plus, Eppendorf).

Phosphopeptide enrichment was performed using a FeCl_3_-charged ProPAC IMAC-10 column (Thermo Scientific) as described previously [6]. Phosphopeptide-enriched fractions were desalted using a 1 cc Oasis HLB cartridge (Waters Corp.). Samples were analyzed on a nanoflow ultrahigh-performance liquid chromatography (UPLC) system (400 Series, Eksigent/Sciex) hyphenated with a quadrupole-Orbitrap mass spectrometer (Q Exactive; Thermo Scientific). The Q Exactive mass spectrometer was run in positive-ion mode. Full scans were carried out at a resolution of 70k with an automatic gain control (AGC) target of 3 x 10^6^ ions and a maximum injection time of 120 ms, using a scan range of 375 to 1400 m/z. For tandem MS (MS/MS) data acquisition, a normalized collision energy value of 27 was used. Scans were carried out at a resolution of 17.5 K with an AGC target of 5 x 10^4^ ions and a maximum injection time of 100 ms. The isolation window was set to 1.6 m/z. An underfill ratio of 2.0% was set and a dynamic exclusion value of 25.0 s applied.

The mass spectrometry proteomics data have been deposited to the ProteomeXchange Consortium via the PRIDE partner repository with the dataset identifier PXD015197. RAW files generated using XCalibur software (version 2.2; Thermo Scientific), were analyzed using MaxQuant (version 1.5.6.5) for identification and quantification of phosphopeptides [60]. Phosphorylation (STY) and oxidation (M) were used as variable modifications and carbamidomethylation as a fixed modification. The “match between run” and “label-free quantification (LFQ)” options were enabled. Search was delimited to the *M*. *tuberculosis* H37RV version 2 protein sequence database, downloaded from PATRIC [61]. Finally, the evidence file was used to extract the identified peptides. Using the modified sequence column, the average intensity of each modified peptide was calculated.

### Differential abundance testing of phosphopeptides between *M*. *tuberculosis* strains

Differential abundance testing of induced versus uninduced kinase expression was done for each strain using the metagenomeSeq package in R [62]. Within each strain, only phosphopeptides with non-zero in intensities in 8 or more out of 18 samples (17 samples for *pknA* depletion because one sample was excluded at the data analysis stage because of poor data quality) were considered for abundance testing. When the same peptide sequence with the same phosphorylation site was detected but differed in either charge state, acetylation, or oxidation, we picked a representative peptide with the highest mean intensity (after median normalizing by sample) across all samples; this representative peptide is shown in the “cleanseq” column in the supplemental tables. The original intensities were then median normalized by sample using only phosphopeptides that met these criteria. For each phosphopeptide, log_2_ fold-change (log_2_FC) values for the depletion versus control samples were then calculated by metagenomeSeq, controlling for batch effects. The Pearson correlations of log_2_ fold-change values between strains were computed using phosphopeptides with log_2_ fold-change values estimated in both strains.

### pLogo generation

For each of the kinase-depleted data sets, significantly increased or decreased Ser- and Thr-containing phosphopeptides were mapped onto the UniProt *M*. *tuberculosis* proteome and extended with adjacent sequence information where necessary. This generated aligned foreground data sets of 15-mers in which the Ser/Thr phosphorylation sites were always located at the central (0) position. Duplicate sequences, sequences with phosphorylated residues within 7 residues of a protein terminus, and sequences for which there exist multiple possible mappings onto the *M*. *tuberculosis* proteome were discarded. The aligned background data set of 15-mers was generated by all Ser and Thr residues in the *M*. *tuberculosis* proteome with their ±7 flanking residues.

These foreground and background data sets were used as inputs to analyze the phosphorylation motifs corresponding to each kinase using the pLogo tool (http://plogo.uconn.edu) [38]. pLogos depict residues proportionally to the log-odds binomial probability of each residue at each position given the residue frequencies in the background. Overrepresented residues are drawn above and underrepresented residues are drawn below the x-axis. The most statistically significant residues are drawn closest to the x-axis, and a red horizontal bar denotes the 0.05 significance level following Bonferroni correction. pLogos centered at Thr and Ser were generated for each data set, except for the decreased Ser-phosphorylated peptides in the *pknA+pknB* depletion strain and the increased Ser-phosphorylated peptides in the *pknB* depletion strain, for which there were too few differentially expressed Ser-phosphorylated peptides to analyze.

### Total lipid extraction, cell wall-bound mycolic acids (MA) isolation, and thin-layer chromatography (TLC)

*M*. *tuberculosis* wild type and all three depletion mutants were cultured in 7H9+ADN medium to early exponential phase. Bacteria were then harvested according to published methods [32]. Briefly, pellets were washed three times in 10 ml distilled water and then resuspended in 15 ml methanol/chloroform (2:1) for 24 hours for sterilization and lipid extraction. The extractable lipids were separated from the cell pellet by centrifugation. The extraction was repeated with another 15 mL of methanol/chloroform (1:2) for one hour. The extracts were combined as the total lipids. The total lipid profiles were analyzed by TLC (Silica Gel 60, Macherey-Nagel) using a solvent system of chloroform/methanol/water (60/16/2). For the cell wall bound mycolic acids, the delipidated cell wall was saponified as previously described [63]. For TLC analysis, the saponified mycolic acids were methylated to yield mycolic acid methyl esters (MAMEs), which were developed three times with a solvent system of hexanes /diethyl ether (90/15) and developed with 8% (v/v) phosphoric acid and 3% (w/v) cupric acetate and charring.

### ^13^C labeling of *M*. *tuberculosis* mycolic acids and mass spectrometry analysis

For ^13^C labeling, culture media was supplemented without or with 3 mg/ml of [1, 2-^13^C] acetate for 48 hours prior to harvest. Total lipids (for TLC analysis) and cell wall-bound mycolic acids were isolated from the delipidated cell walls as described above. Mycolic acids were analyzed by HPLC-ESI-MS (Agilent 6530 Accurate-Mass Q-TOF and 1260 series HPLC system using a normal phase Varian MonoChrom Diol column) [64]. The isocratic HPLC mobile phase solvents were hexane/isopropyl alcohol (70:30 (v:v)), supplemented with formic acid 0.1% (v:v) and ammonium hydroxide 0.05% (v:v) and samples were run at 0.15 ml/min for 10 min. Collision-induced dissociation mass spectrometry (CID-MS) was carried-out with a collision energy of 40 V and the isolation width was set to 4 m/z. For the ^13^C labeled sample, the precursor ions located in the labeling area 1192 m/z was chosen for the CID-MS.

## Supporting information

S1 Table Primers, vectors and bacterial strains. (XLSX)

S2 Table Total and unique phosphoproteins and phosphopeptides. All quantified phosphopeptides (phosphopeptides present in at least 8 of 18 samples for each strain) and the proteins that contain these peptides.
(XLSX)

S3 Table Significant differential phosphorylation in each strain. Phosphopeptides that have significantly increased or decreased phosphorylation in each kinase depletion strain.
(XLSX)

S4 Table Differentially phosphorylated peptides and candidate substrate assignment. Candidate PknA substrate phosphorylation sites are defined as showing significantly decreased phosphorylation in the *pknA* depletion strain and either no significant change or significantly increased phosphorylation or phosphorylation in the *pknB* depletion strain. Candidate PknB substrate phosphorylation sites are defined as showing significantly decreased phosphorylation in the *pknB* depletion strain and either no significant change or significantly increased phosphorylation or phosphorylation in the *pknA* depletion strain. Candidate substrate phosphorylation sites of both PknA and PknB are defined as showing significantly decreased phosphorylation in both the *pknA* depletion strain and the *pknB* depletion strain.
(XLSX)

S1 Fig Strain construction and expression of *pknA* and *pknB* in the *pknA*, *pknA*+ *pknB*, and *pknB* depletion strains. **S1A)** Schematic representation of the kinase depletion mutant construction. The *pknA*+*pknB* depletion strain was obtained by single crossover homologous recombination of the amino-terminal region of *pknA* under control of the pristinamycin (ptc)-inducible pptr promoter into the chromosomal copy of *pknA* [25]. The position of *pknB* immediately 3’ of *pknA*, places both *pknA* and *pknB* under control of pptR. To obtain a *pknA* only depletion strain, *pknB* under the control of a TetR-regulated promoter [53] was integrated at separate site (mycobacteriophage L5 *attB* site [65]) in the *pknA+pknB* depletion strain, so that *pknB* expression can be induced with atc. Recombination of the 5’ region of *pknB* under control of the pptR promoter into the chromosomal copy of *pknB* was used to create a strain in which *pknB* alone can be depleted. **S1B)** A tetracycline repressor (TetR)-regulated copy of *pknB* was introduced into the *pknA+pknB* conditional depletion strain. In the absence of ptc or atc induction both *pknA* and *pknB* are depleted, whereas induction with ptc induces expression of both genes. When atc is added in the absence of ptc, only *pknA* is depleted. S1C) Wild type, *pknA*+*pknB* depletion, *pknB* depletion and *pknA* depletion strains were grown in broth and plated on 7H9 agar with or without ptc and atc as indicated and incubated for 3 weeks, demonstrating the absence of growth when *pknA* alone, *pknB* alone or both *pknA* and *pknB* are depleted.
(TIF)

S2 Fig Viability over time of wild type and kinase depletion strains. Each strain was grown in Middlebrook 7H9-ADN-Tw broth to OD_600_ = 0.6, diluted back to OD_600_ = 0.02, followed by growth in medium with and without ptc or atc as indicated. Serial dilutions were plated at days 4, 8 and 12 colonies were counted after 3 weeks incubation. Day 0 counts were not available for this experiment, but the available data show decreased CFU from days 4 to 12 for each depletion strain.
(TIF)

S3 Fig Analysis of total and newly synthesized fatty accids in wild type and kinase depletion strains. **S3A)** Thin layer chromatography of saponified cell wall mycolic acids of *M*. *tuberculosis* from wild type and kinase depletion strains. *M*. *tuberculosis* (wild type), *pknA*, *pknB*, *pknA+B* depletion strains were grown with 0.25 μg/ml pristinamycin at 37°C in 7H9+AND+tw until they reached an OD_600_ of 0.8. The cells were spun down, washed with PBS-Tx (PBS plus 0.05% tyloxapol), and diluted to an OD_600_ of 0.1, and then grown +/-0.25 μg/ml pristinamycin in 7H9+AND without tween-80. At each time point, cells were pelleted and washed with PBS. Biological duplicate samples for TLC were harvested at serial time points by resuspending the bacteria into 15 mL 1:2 (V:V) chloroform:methanol to sterilize samples and extract lipids. Total mycolates were isolated by saponification as previously described [32], dried down under nitrogen and analyzed by TLC (Silica Gel 60, Macherey-Nagel) using 3 developments with 90:15 (v/v) hexane: diethyl ether and developed with 8% (v/v) phosphoric acid and 3% (w/v) cupric acetate and charring. **S3B)** Detection of newly synthesized fatty acids using ^13^C labeling of wild type and kinase depletion strains. HPLC-MS negative ion mode analysis of the free fatty acid (m/z 395.39) was performed on total lipid extracts from the wild type and kinase depletion strains to measure ^13^C incorporation from [1,2-^13^C] acetate uptake. The ^13^C labeling gave additional peaks from m/z 400 to 415 for all strains. The total lipid extracts used here were also used in experiments shown in Fig 5E and 5F [64].
(TIF)

S4 Fig Potentiation of cefotaxime and meropenem in the *pknA* depletion strain with *pknB* expression induced by two different atc concentrations. Inhibitory concentrations of cefotaxime and meropenem in wild type and in the *pknA* depletion strain were determined using the MABA assay as described in the Materials and Methods section. Experiments were performed with *pknB* expression induced by either 10 or 20 ng/ml of atc. **S4A)** Representative images of wild type and *pknA* depletion strains tested for meropenem susceptibility. The - and + signs above the columns at the left side of each image indicate negative control (medium) and positive control (growth in the absence of antibiotic), respectively. The numbers in red indicate the ptc concentration in μg/ml in each row or column. The yellow circle in the positive control column in the lower panels indicates the well with the lowest ptc concentration that allowed growth in the absence of antibiotics (0.008 μg/ml ptc). The yellow circle in the 0.008 μg/ml column on the right side of the lower images indicates the lowest meropenem concentration that inhibited growth at this ptc concentration. **S4B)** Summary table of results for wild type and *pknA* depletion strains with cefotaxime and meropenem. The lowest inhibitory concentration in the *pknA* depletion strain is the same when 10 or 20 ng/ml of atc is used and is shown as ≤ 0.25 μg/ml because this was the lowest concentration of antibiotics included in these experiments. Biological duplicate experiments were performed for all strains.
(TIF)

S5 Fig Acid-fast staining of the *pknA* depletion strain with *pknB* expression induced by two different atc concentrations. Acid-fast staining was performed as described in the materials and methods section on wild type and *pknA* depletion strains grown in Middlebrook 7H9 medium. The *pknA* depletion strain was grown in the presence of ptc to induce expression of both *pknA* and *pknB*, or in the presence of 10 ng/ml or 20 ng/ml atc to induce the expression of *pknB* but not *pknA*. The wild type and ptc-induced *pknA* depletion strain show acid fast staining of most cells (bright pink), while most cells the *pknA* depletion strain grown in the presence of 10 or 20 ng/ml of atc are not acid-fast and stain blue with the counterstain.
(TIF)

S6 Fig Growth curves of *M*. *tuberculosis* (wild type), *pknAB* depletion, *pknB* depletion mutant, *pknA* depletion mutant used for phosphoproteomic analyses. The cells were grown with 0.25 μg/ml pristinamycin until they reached an OD_600_ of 0.8. The cells were spun down, washed with PBS-Tx (PBS plus 0.05% tyloxapol), and diluted to an OD_600_ of 0.1, and then grown +/- 0.25 μg/ml pristinamycin in 7H9+AND, plus induction of *pknB* by 20ng/ml atc for *pknA* depletion. OD_600_ measurements were performed every 24h. Note the slower onset of growth arrest for the *pknB* depletion strain in these cultures with a 10-fold higher inoculum compared to Fig 3. Data are the average of three biological replicates and error bars represent +/- 1 SD.
(TIF)

S7 Fig Comparison of differential abundance of phosphopeptides from the three depletion strains. **S7A.** Venn diagram of the significantly decreased unique phosphopeptides identified in each strain. **S7B.** Venn diagram of the significantly increased unique phosphopeptides identified in each strain. **S7C-E**. Pearson correlation analysis of Log_2_FC of phosphopeptides in kinase-depleted relative to kinase replete bacteria from each of the three depletion strains. Each dot represents a phosphopeptide that was quantified in both of the strains being compared **S7C.** *pknA* Log_2_FC vs. *pknB* Log_2_FC, **S7D.** *pknB* Log_2_FC vs. *pknAB* Log_2_FC, **S7E.** *pknA* Log_2_FC vs. *pknAB* Log_2_FC.
(TIF)

S8 Fig Venn diagrams of overlap between phosphoproteins from the current and prior studies. **S8A**) Overlap of the decreased phosphoproteins identified in *pknB* depletion data from this study (N = 43, red) compared to phosphoproteins with decreased phosphorylation identified in *pknB* depletion data from Turapov et al (N = 13, blue) [24]. **S8B)** Overlap of the decreased phosphoproteins identified in the *pknA*+*pknB* depletion data from this study (N = 60, red) compared to decreased phosphoproteins identified in response to treatment with a small molecule chemical inhibitor of both PknA and PknB (Carette, N = 48, blue) [6]. **S8C)** Overlap of the decreased phosphoproteins identified in this study in *pknA* depletion (N = 171, red), *pknB* depletion (green, n = 43), and *pknA*+*pknB* depletion (yellow, n = 60) strains compared to proteins showing decreased phosphorylation in response to small molecule inhibition of PknA and PknB in Carette, et al (N = 48, blue). **S8D)** Overlap of all quantified phosphoproteins in this study (N = 712, blue) compared to two prior phosphoproteomic studies from this laboratory, Carette 2018 (all quantified phosphoproteins, N = 417, red), and a study that identified phosphorylation sites in *M*. *tuberculosis* proteins at different growth stages and following exposure to stresses (Prisic 2010, N = 301, green) [6, 7].
(TIF)

S9 Fig **S9A**, pLogos showing relative statistical significance of amino acids at positions adjacent to the phosphoacceptor Ser in phosphopeptides that were significantly decreased in the *pknA* or *pknB* depletion strains. **S9B**, pLogos showing relative statistical significance of amino acids at positions adjacent to the phosphoacceptor Thr in phosphopeptides that were significantly increased in each kinase depletion strain. **S9C**, pLogos showing relative statistical significance of amino acids at positions adjacent to the phosphoacceptor Ser in phosphopeptides that were significantly increased in *pknA* or *pknA*+*pknB* kinase depletion strain (there were too few increased Ser-phosphorylated peptides in the *pknB*-depletion strain to analyze). The red horizontal line indicates P = 0.05 after Bonferroni correction.
(TIF)

## References

[1] World Health Organization. Global Tuberculosis Report 2019. Geneva: World Health Organization 2019.

[2] Koch A, Cox H, Mizrahi V. Drug-resistant tuberculosis: challenges and opportunities for diagnosis and treatment. Current Opinion in pharmacology. 2018;42:7–15. 10.1016/j.coph.2018.05.013 . 29885623 PMC6219890

[3] Behr MA, Edelstein PH, Ramakrishnan L. Revisiting the timetable of tuberculosis. BMJ. 2018;362:k2738 Epub 2018/08/25. 10.1136/bmj.k2738 . 30139910 PMC6105930

[4] Wehenkel A, Bellinzoni M, Graña M, Duran R. Mycobacterial Ser/Thr protein kinases and phosphatases: physiological roles and therapeutic potential. Biochim Biophys Acta. 2008;1784:193–202. 15234634935205355210related:yt6ry8FLbNMJ. 10.1016/j.bbapap.2007.08.006 17869195

[5] Av-Gay Y, Everett M. The eukaryotic-like Ser/Thr protein kinases of *Mycobacterium tuberculosis*. Trends Microbiol. 2000;8(5):238–44. 10.1016/s0966-842x(00)01734-0 10785641

[6] Carette X, Platig J, Young DC, Helmel M, Young AT, Wang Z, et al Multisystem Analysis of *Mycobacterium tuberculosis* Reveals Kinase-Dependent Remodeling of the Pathogen-Environment Interface. mBio. 2018;9(2):e02333–17. Epub 2018/03/08. 10.1128/mBio.02333-17 . 29511081 PMC5845002

[7] Prisic S, Dankwa S, Schwartz D, Chou MF, Locasale JW, Kang CM, et al Extensive phosphorylation with overlapping specificity by *Mycobacterium tuberculosis* serine/threonine protein kinases. Proc Natl Acad Sci U S A. 2010;107(16):7521–6. 10.1073/pnas.0913482107 . 20368441 PMC2867705

[8] Kang CM, Abbott DW, Park ST, Dascher CC, Cantley LC, Husson RN. The *Mycobacterium tuberculosis* serine/threonine kinases PknA and PknB: substrate identification and regulation of cell shape. Genes Dev. 2005;19:1692–704. 10.1101/gad.1311105 . 15985609 PMC1176007

[9] Kusebauch U, Ortega C, Ollodart A, Rogers RS, Sherman DR, Moritz RL, et al *Mycobacterium tuberculosis* supports protein tyrosine phosphorylation. Proc Natl Acad Sci U S A. 2014;111(25):9265–70. 10.1073/pnas.1323894111 . 24927537 PMC4078798

[10] Gee CL, Papavinasasundaram KG, Blair SR, Baer CE, Falick AM, King DS, et al A phosphorylated pseudokinase complex controls cell wall synthesis in mycobacteria. Science Signaling. 2012;5(208):ra7 Epub 2012/01/26. 10.1126/scisignal.2002525 . 22275220 PMC3664666

[11] Wu P, Nielsen TE, Clausen MH. Small-molecule kinase inhibitors: an analysis of FDA-approved drugs. Drug Discov Today. 2016;21(1):5–10. 10.1016/j.drudis.2015.07.008 58016775481705154related:wsKZLfMdzgAJ. 26210956

[12] Hanks S, Hunter T. The eukaryotic protein kinase superfamily: kinase (catalytic) domain structure and classification. FASEB J. 1995;9:576–96. 7768349

[13] Sassetti CM, Boyd DH, Rubin EJ. Genes required for mycobacterial growth defined by high density mutagenesis. Mol Microbiol. 2003;48(1):77–84. 10.1046/j.1365-2958.2003.03425.x . 12657046

[14] DeJesus MA, Gerrick ER, Xu W, Park SW, Long JE, Boutte CC, et al Comprehensive Essentiality Analysis of the *Mycobacterium tuberculosis* Genome via Saturating Transposon Mutagenesis. MBio. 2017;8(1). Epub 2017/01/18. 10.1128/mBio.02133-16 . 28096490 PMC5241402

[15] Nagarajan SN, Upadhyay S, Chawla Y, Khan S, Naz S, Subramanian J, et al Protein kinase A (PknA) of *Mycobacterium tuberculosis* is independently activated and is critical for growth in vitro and survival of the pathogen in the host. J Biol Chem. 2015;290(15):9626–45. 10.1074/jbc.M114.611822 . 25713147 PMC4392265

[16] Chawla Y, Upadhyay SK, Khan S, Nagarajan SN, Forti F, Nandicoori VK. Protein Kinase B (PknB) of *Mycobacterium tuberculosis* is essential for growth of the pathogen in vitro as well as for survival within the host. J Biol Chem. 2014;289(20):13858–75. 10.1074/jbc.M114.563536 . 24706757 PMC4022859

[17] Prisic S, Husson RN. *Mycobacterium tuberculosis* Serine/Threonine Protein Kinases. Microbiol Spectr. 2014;2(5). 10.1128/microbiolspec.MGM2-0006-2013 . 25429354 PMC4242435

[18] Mir M, Asong J, Li X, Cardot J, Boons GJ, Husson RN. The Extracytoplasmic Domain of the *Mycobacterium tuberculosis* Ser/Thr Kinase PknB Binds Specific Muropeptides and Is Required for PknB Localization. PLoS Pathogens. 2011;7(7):e1002182 Epub 2011/08/11. 10.1371/journal.ppat.1002182 . 21829358 PMC3145798

[19] Vilchèze C, Molle V, Carrère-Kremer S, Leiba J, Mourey L, Shenai S, et al Phosphorylation of KasB regulates virulence and acid-fastness in *Mycobacterium tuberculosis*. PLoS Pathogens. 2014;10(5):e1004115 10.1371/journal.ppat.1004115 . 24809459 PMC4014462

[20] Corrales RM, Molle V, Leiba J, Mourey L, de Chastellier C, Kremer L. Phosphorylation of Mycobacterial PcaA Inhibits Mycolic Acid Cyclopropanation: Consequences for intracellular survival and for phagosome maturation block. J Biol Chem. 2012;287(31):26187–99. 10.1074/jbc.M112.373209 . 22621931 PMC3406704

[21] Boutte CC, Baer CE, Papavinasasundaram K, Liu W, Chase MR, Méniche X, et al A cytoplasmic peptidoglycan amidase homologue controls mycobacterial cell wall synthesis. Elife. 2016;5 10.7554/eLife.14590 . 27304077 PMC4946905

[22] Ventura M, Rieck B, Boldrin F, Degiacomi G, Bellinzoni M, Barilone N, et al GarA is an essential regulator of metabolism in *Mycobacterium tuberculosis*. Mol Microbiol. 2013;90(2):356–66. 10.1111/mmi.12368 . 23962235

[23] Sajid A, Arora G, Gupta M, Singhal A, Chakraborty K, Nandicoori VK, et al Interaction of *Mycobacterium tuberculosis* elongation factor Tu with GTP is regulated by phosphorylation. J Bacteriol. 2011;193(19):5347–58. 10.1128/JB.05469-11 . 21803988 PMC3187401

[24] Turapov O, Forti F, Kadhim B, Ghisotti D, Sassine J, Straatman-Iwanowska A, et al Two Faces of CwlM, an Essential PknB Substrate, in *Mycobacterium tuberculosis*. Cell Reports. 2018;25(1):57–67.e5. 10.1016/j.celrep.2018.09.004 . 30282038 PMC6180346

[25] Forti F, Crosta A, Ghisotti D. Pristinamycin-inducible gene regulation in mycobacteria. J of biotechnology. 2009;140(3–4):270–7. 10.1016/j.jbiotec.2009.02.001 . 19428723

[26] Carroll P, Schreuder LJ, Muwanguzi-Karugaba J, Wiles S, Robertson BD, Ripoll J, et al Sensitive detection of gene expression in mycobacteria under replicating and non-replicating conditions using optimized far-red reporters. PLoS ONE. 2010;5(3):e9823 10.1371/journal.pone.0009823 . 20352111 PMC2843721

[27] Pandey AK, Raman S, Proff R, Joshi S, Kang CM, Rubin EJ, et al Nitrile-inducible gene expression in mycobacteria. Tuberculosis (Edinb). 2008 10.1016/j.tube.2008.07.007 . 18801704 PMC2845969

[28] Rego EH, Audette RE, Rubin EJ. Deletion of a mycobacterial divisome factor collapses single-cell phenotypic heterogeneity. Nature. 2017;546(7656):153–7. 10.1038/nature22361 . 28569798 PMC5567998

[29] Kang C-M, Nyayapathy S, Lee J-Y, Suh J-W, Husson RN. Wag31, a homologue of the cell division protein DivIVA, regulates growth, morphology and polar cell wall synthesis in mycobacteria. Microbiology (Reading, England). 2008;154(Pt 3):725–35. 10.1099/mic.0.2007/014076-0 . 18310019

[30] Méniche X, Otten R, Siegrist MS, Baer CE, Murphy KC, Bertozzi CR, et al Subpolar addition of new cell wall is directed by DivIVA in mycobacteria. Proc Natl Acad Sci U S A. 2014 10.1073/pnas.1402158111 . 25049412 PMC4128124

[31] Bhatt A, Molle V, Besra GS, Jacobs WR Jr, Kremer L. The *Mycobacterium tuberculosis* FAS-II condensing enzymes: their role in mycolic acid biosynthesis, acid-fastness, pathogenesis and in future drug development. Molecular Microbiology. 2007;64(6):1442–54. 10.1111/j.1365-2958.2007.05761.x 17555433

[32] Lehmann J, Cheng T-Y, Aggarwal A, Park AS, Zeiler E, Raju RM, et al An Antibacterial β-Lactone kills *Mycobacterium tuberculosis* by disrupting mycolic acid synthesis. Angewandte Chemie. 2017;130(1):354–9. 10.1002/ange.201709365 PMC6104829 29067779

[33] Xu Z, Meshcheryakov VA, Poce G, Chng S-S. MmpL3 is the flippase for mycolic acids in mycobacteria. Proc Natl Acad Sci U S A. 2017;114(30):7993–8. 10.1073/pnas.1700062114 . 28698380 PMC5544280

[34] Kristich CJ, Wells CL, Dunny GM. A eukaryotic-type Ser/Thr kinase in *Enterococcus faecalis* mediates antimicrobial resistance and intestinal persistence. Proc Natl Acad Sci USA. 2007;104(9):3508–13. 10.1073/pnas.0608742104 17360674 PMC1805595

[35] Pensinger DA, Aliota MT, Schaenzer AJ, Boldon KM, Ansari I-uH, Vincent WJB, et al Selective pharmacologic inhibition of a PASTA kinase increases *Listeria monocytogenes* susceptibility to β-lactam antibiotics. Antimicrobial Agents and Chemotherapy. 2014;58(8):4486–94. 10.1128/AAC.02396-14 . 24867981 PMC4135996

[36] Tamber S, Schwartzman J, Cheung AL. Role of PknB kinase in antibiotic resistance and virulence in community-acquired methicillin-resistant *Staphylococcus aureus* strain USA300. Infection and immunity. 2010;78(8):3637–46. 10.1128/IAI.00296-10 . 20547748 PMC2916262

[37] Wlodarchak N, Teachout N, Beczkiewicz J, Procknow R, Schaenzer AJ, Satyshur K, et al In Silico Screen and Structural Analysis Identifies Bacterial Kinase Inhibitors which Act with β-Lactams To Inhibit Mycobacterial Growth. Molecular Pharmaceutics. 2018;15(11):5410–26. 10.1021/acs.molpharmaceut.8b00905 . 30285456 PMC6648700

[38] O’Shea JP, Chou MF, Quader SA, Ryan JK, Church GM, Schwartz D. pLogo: a probabilistic approach to visualizing sequence motifs. Nature Methods. 2013;10(12):1211–2. 10.1038/nmeth.2646 . 24097270

[39] Purushotham G, Sarva KB, Blaszczyk E, Rajagopalan M, Madiraju MV. *Mycobacterium tuberculosis oriC* sequestration by MtrA response regulator. Mol Microbiol. 2015;98(3):586–604. 10.1111/mmi.13144 . 26207528 PMC4700885

[40] Brocker M, Mack C, Bott M. Target genes, consensus binding site, and role of phosphorylation for the response regulator MtrA of *Corynebacterium glutamicum*. J Bacteriol. 2011;193(5):1237–49. 10.1128/JB.01032-10 . 21183673 PMC3067596

[41] Rajagopalan M, Dziedzic R, Al Zayer M, Stankowska D, Ouimet M-C, Bastedo DP, et al *Mycobacterium tuberculosis* origin of replication and the promoter for immunodominant secreted antigen 85B are the targets of MtrA, the essential response regulator. J Biol Chem. 2010;285(21):15816–27. 10.1074/jbc.M109.040097 . 20223818 PMC2871449

[42] Cruz JW, Sharp JD, Hoffer ED, Maehigashi T, Vvedenskaya IO, Konkimalla A, et al Growth-regulating *Mycobacterium tuberculosis* VapC-mt4 toxin is an isoacceptor-specific tRNase. Nature Communications. 2015;6:7480–. 10.1038/ncomms8480 . 26158745 PMC4620994

[43] Schifano JM, Edifor R, Sharp JD, Ouyang M, Konkimalla A, Husson RN, et al Mycobacterial toxin MazF-mt6 inhibits translation through cleavage of 23S rRNA at the ribosomal A site. Proc Natl Acad Sci U S A. 2013;110(21):8501–6. 10.1073/pnas.1222031110 . 23650345 PMC3666664

[44] Harms A, Maisonneuve E, Gerdes K. Mechanisms of bacterial persistence during stress and antibiotic exposure. Science. 2016;354(6318). 10.1126/science.aaf4268 . 27980159

[45] Shah IM, Dworkin J. Induction and regulation of a secreted peptidoglycan hydrolase by a membrane Ser/Thr kinase that detects muropeptides. Mol Microbiol. 2010;75:1232–43. 10.1111/j.1365-2958.2010.07046.x . 20070526

[46] Fenton AK, Manuse S, Flores-Kim J, Garcia PS, Mercy C, Grangeasse C, et al Phosphorylation-dependent activation of the cell wall synthase PBP2a in *Streptococcus pneumoniae* by MacP. Proc Natl Acad Sci U S A. 2018;1:201715218. 10.1073/pnas.1715218115 . 29487215 PMC5856526

[47] Baer CE, Iavarone AT, Alber T, Sassetti CM. Biochemical and spatial coincidence in the provisional Ser/Thr protein kinase interaction network of *Mycobacterium tuberculosis*. J Biol Chem. 2014;289(30):20422–33. 10.1074/jbc.M114.559054 . 24928517 PMC4110253

[48] Wagner T, Alexandre M, Duran R, Barilone N, Wehenkel A, Alzari PM, et al The crystal structure of the catalytic domain of the ser/thr kinase PknA from *M*. *tuberculosis* shows an Src-like autoinhibited conformation. Proteins. 2015;83(5):982–8. 10.1002/prot.24754 . 25586004

[49] Ortega C, Liao R, Anderson LN, Rustad T, Ollodart AR, Wright AT, et al *Mycobacterium tuberculosis* Ser/Thr protein kinase B mediates an oxygen-dependent replication switch. PLoS Biology. 2014;12(1):e1001746 10.1371/journal.pbio.1001746 . 24409094 PMC3883633

[50] Jani C, Eoh H, Lee JJ, Hamasha K, Sahana MB, Han J-S, et al Regulation of polar peptidoglycan biosynthesis by Wag31 phosphorylation in mycobacteria. BMC Microbiology. 2010;10:327 10.1186/1471-2180-10-327 . 21190553 PMC3019181

[51] Goldstein BP. Resistance to rifampicin: a review. J Antibiot. 2014;67(9):625–30. papers3://publication/ 10.1038/ja.2014.107 25118103

[52] Shell SS, Wang J, Lapierre P, Mir M, Chase MR, Pyle MM, et al Leaderless Transcripts and Small Proteins Are Common Features of the Mycobacterial Translational Landscape. PLoS Genet. 2015;11(11):e1005641 10.1371/journal.pgen.1005641 . 26536359 PMC4633059

[53] Klotzsche M, Ehrt S, Schnappinger D. Improved tetracycline repressors for gene silencing in mycobacteria. Nucleic Acids Res. 2009;37(6):1778–88. 10.1093/nar/gkp015 19174563 PMC2665214

[54] Blokpoel MC, Murphy HN, O'Toole R, Wiles S, Runn ES, Stewart GR, et al Tetracycline-inducible gene regulation in mycobacteria. Nucleic Acids Res. 2005;33(2):e22 10.1093/nar/gni023 . 15687380 PMC548381

[55] Sampson SL, Dascher CC, Sambandamurthy VK, Russell RG, Jacobs WR, Bloom BR, et al Protection elicited by a double leucine and pantothenate auxotroph of *Mycobacterium tuberculosis* in guinea pigs. Infect Immun. 2004;72(5):3031–7. 10.1128/IAI.72.5.3031-3037.2004 15102816 PMC387862

[56] Singh AK, Carette X, Potluri L-P, Sharp JD, Xu R, Prisic S, et al Investigating essential gene function in *Mycobacterium tuberculosis* using an efficient CRISPR interference system. Nucleic Acids Research. 2016:gkw625. 10.1093/nar/gkw625 . 27407107 PMC5062980

[57] Arany Z. High-Throughput Quantitative Real-Time PCR in Current Protocols in Human Genetics. Hoboken, NJ, USA: John Wiley & Sons, Inc.; 2001. 10.1002/0471142905.hg1110s58 18633974

[58] Franzblau SG, Witzig RS, McLaughlin JC, Torres P, Madico G, Hernandez A, et al Rapid, low-technology MIC determination with clinical *Mycobacterium tuberculosis* isolates by using the microplate Alamar Blue assay. Journal of clinical microbiology. 1998;36(2):362–6. . 9466742 10.1128/jcm.36.2.362-366.1998 PMC104543

[59] Wiśniewski JR, Zougman A, Nagaraj N, Mann M. Universal sample preparation method for proteome analysis. Nat Meth. 2009;6(5):359–62. 10.1038/nmeth.1322 . 19377485

[60] Cox J, Mann M. MaxQuant enables high peptide identification rates, individualized p.p.b.-range mass accuracies and proteome-wide protein quantification. Nat Biotechnol. 2008;26(12):1367–72. 10.1038/nbt.1511 . 19029910

[61] Wattam AR, Abraham D, Dalay O, Disz TL, Driscoll T, Gabbard JL, et al PATRIC, the bacterial bioinformatics database and analysis resource. Nucleic Acids Res. 2014;42(Database issue):D581–91. 10.1093/nar/gkt1099 . 24225323 PMC3965095

[62] Paulson JN, Stine OC, Bravo HC, Pop M. Differential abundance analysis for microbial marker-gene surveys. Nat Meth. 2013;10(12):1200–2. 10.1038/nmeth.2658 . 24076764 PMC4010126

[63] Hong S, Cheng T-Y, Layre E, Sweet L, Young DC, Posey JE, et al Ultralong C100 mycolic acids support the assignment of *Segniliparus* as a new bacterial genus. PLoS ONE. 2012;7(6):e39017 10.1371/journal.pone.0039017 . 22720018 PMC3375245

[64] Layre E, Sweet L, Hong S, Madigan CA, Desjardins D, Young DC, et al A comparative lipidomics platform for chemotaxonomic analysis of *Mycobacterium tuberculosis*. Chem Biol. 2011;18(12):1537–49. 10.1016/j.chembiol.2011.10.013 . 22195556 PMC3407843

[65] Lee M, Pascopella L, Jacobs W Jr., Hatfull G. Site-specific integration of mycobacteriophage L5: integration-proficient vectors for *Mycobacterium smegmatis*, *Mycobacterium tuberculosis* and bacille Calmette-Guerin. Proc Natl Acad Sci USA. 1991;88:3111–5. 10.1073/pnas.88.8.3111 1901654 PMC51395

