# Supplementary figures and images for: Protein kinases PknA and PknB independently and coordinately regulate essential Mycobacterium tuberculosis physiologies and antimicrobial susceptibility
Source: PLoS Pathog. 2020 Apr 7;16(4):e1008452. doi: 10.1371/journal.ppat.1008452 (PMC7164672; doi:10.1371/journal.ppat.1008452)

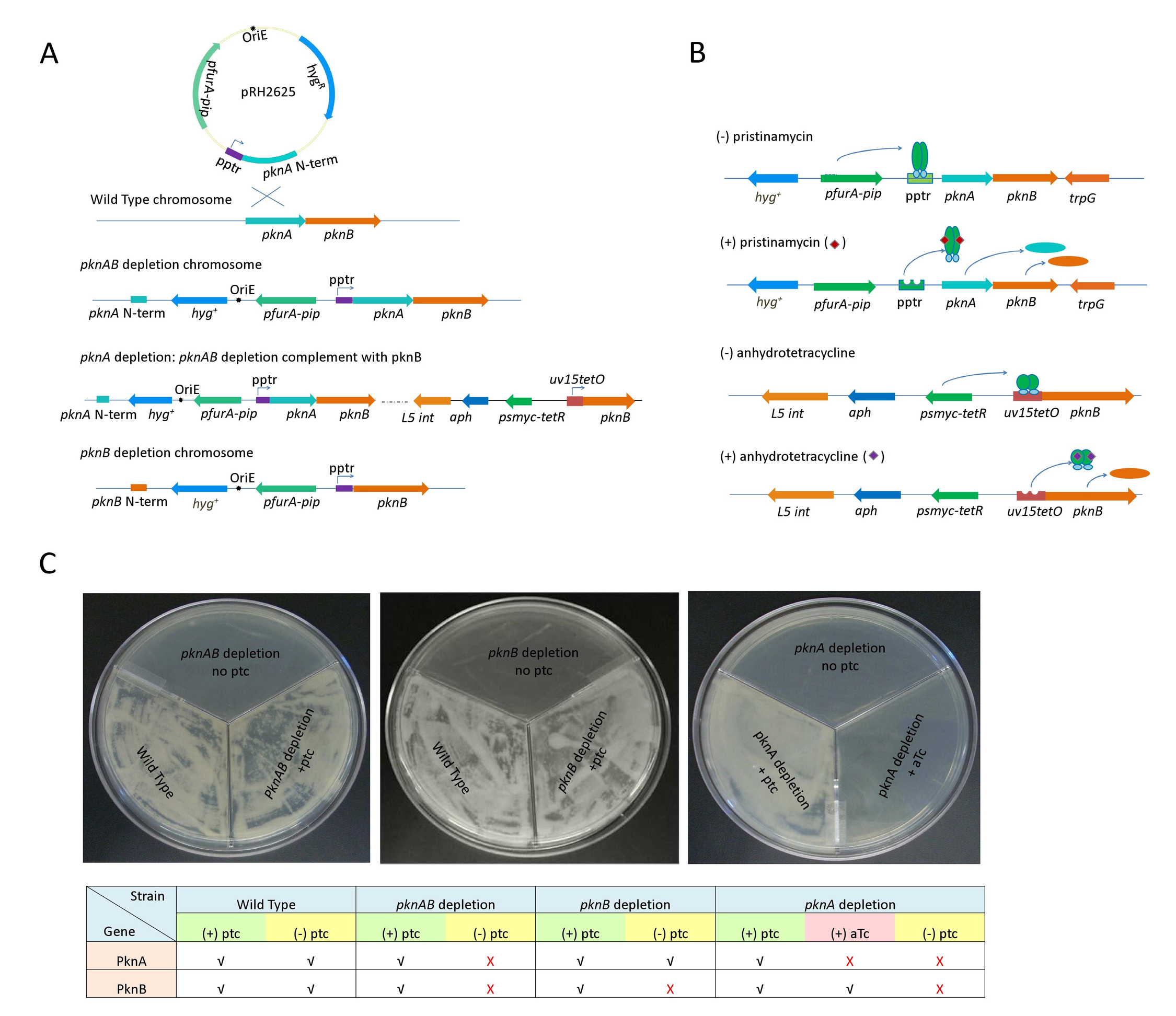

Supplement: S1 Fig — S1A) Schematic representation of the kinase depletion mutant construction. The pknA+pknB depletion strain was obtained by single crossover homologous recombination of the amino-terminal region of pknA under control of the pristinamycin (ptc)-inducible pptr promoter into the chromosomal copy of pknA [25]. The position of pknB immediately 3’ of pknA, places both pknA and pknB under control of pptR. To obtain a pknA only depletion strain, pknB under the control of a TetR-regulated promoter [53] was integrated at separate site (mycobacteriophage L5 attB site [65]) in the pknA+pknB depletion strain, so that pknB expression can be induced with atc. Recombination of the 5’ region of pknB under control of the pptR promoter into the chromosomal copy of pknB was used to create a strain in which pknB alone can be depleted. S1B) A tetracycline repressor (TetR)-regulated copy of pknB was introduced into the pknA+pknB conditional depletion strain. In the absence of ptc or atc induction both pknA and pknB are depleted, whereas induction with ptc induces expression of both genes. When atc is added in the absence of ptc, only pknA is depleted. S1C) Wild type, pknA+pknB depletion, pknB depletion and pknA depletion strains were grown in broth and plated on 7H9 agar with or without ptc and atc as indicated and incubated for 3 weeks, demonstrating the absence of growth when pknA alone, pknB alone or both pknA and pknB are depleted. (TIF) [file ppat.1008452.s005.tif]

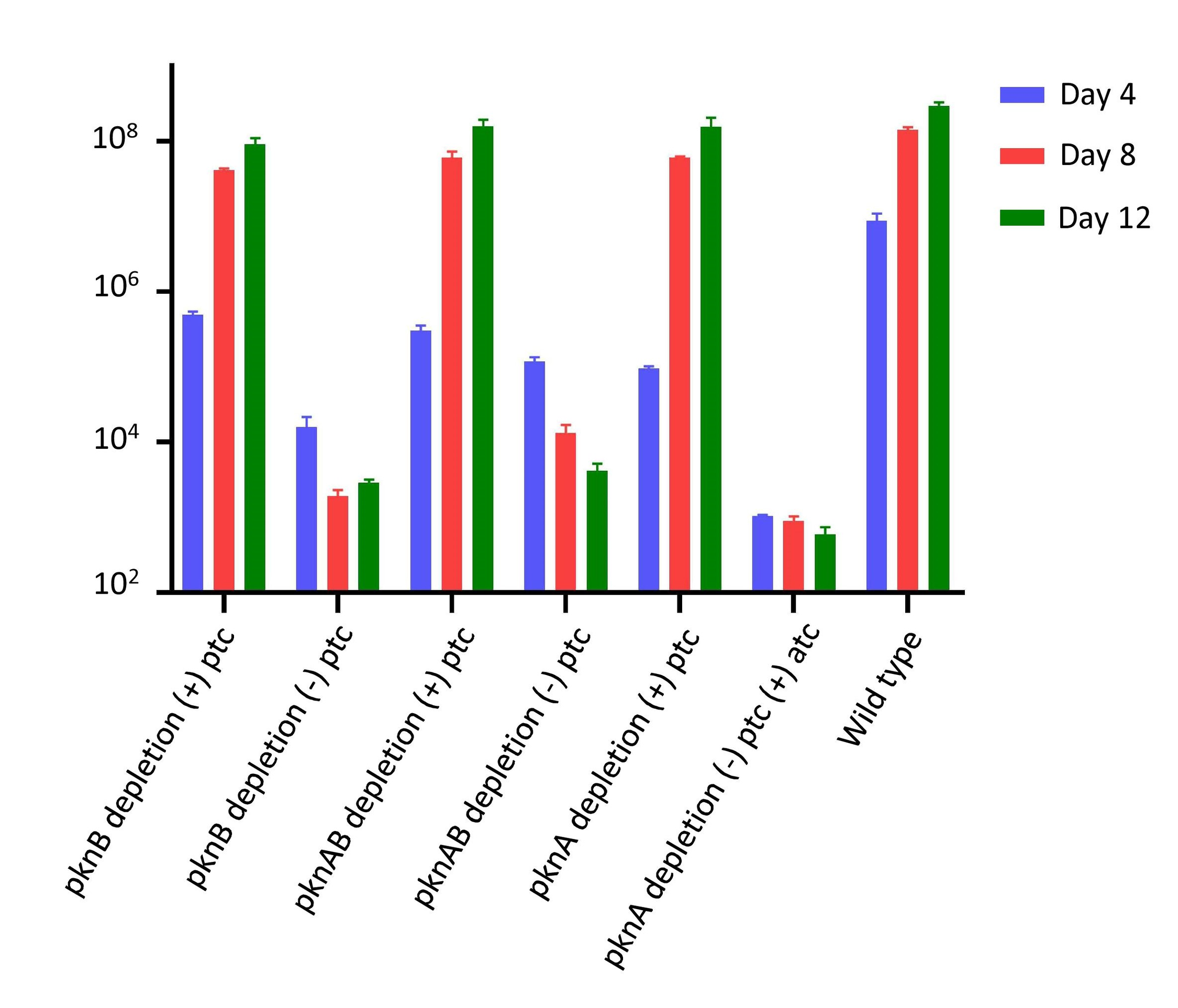

Supplement: S2 Fig — Each strain was grown in Middlebrook 7H9-ADN-Tw broth to OD600 = 0.6, diluted back to OD600 = 0.02, followed by growth in medium with and without ptc or atc as indicated. Serial dilutions were plated at days 4, 8 and 12 colonies were counted after 3 weeks incubation. Day 0 counts were not available for this experiment, but the available data show decreased CFU from days 4 to 12 for each depletion strain. (TIF) [file ppat.1008452.s006.tif]

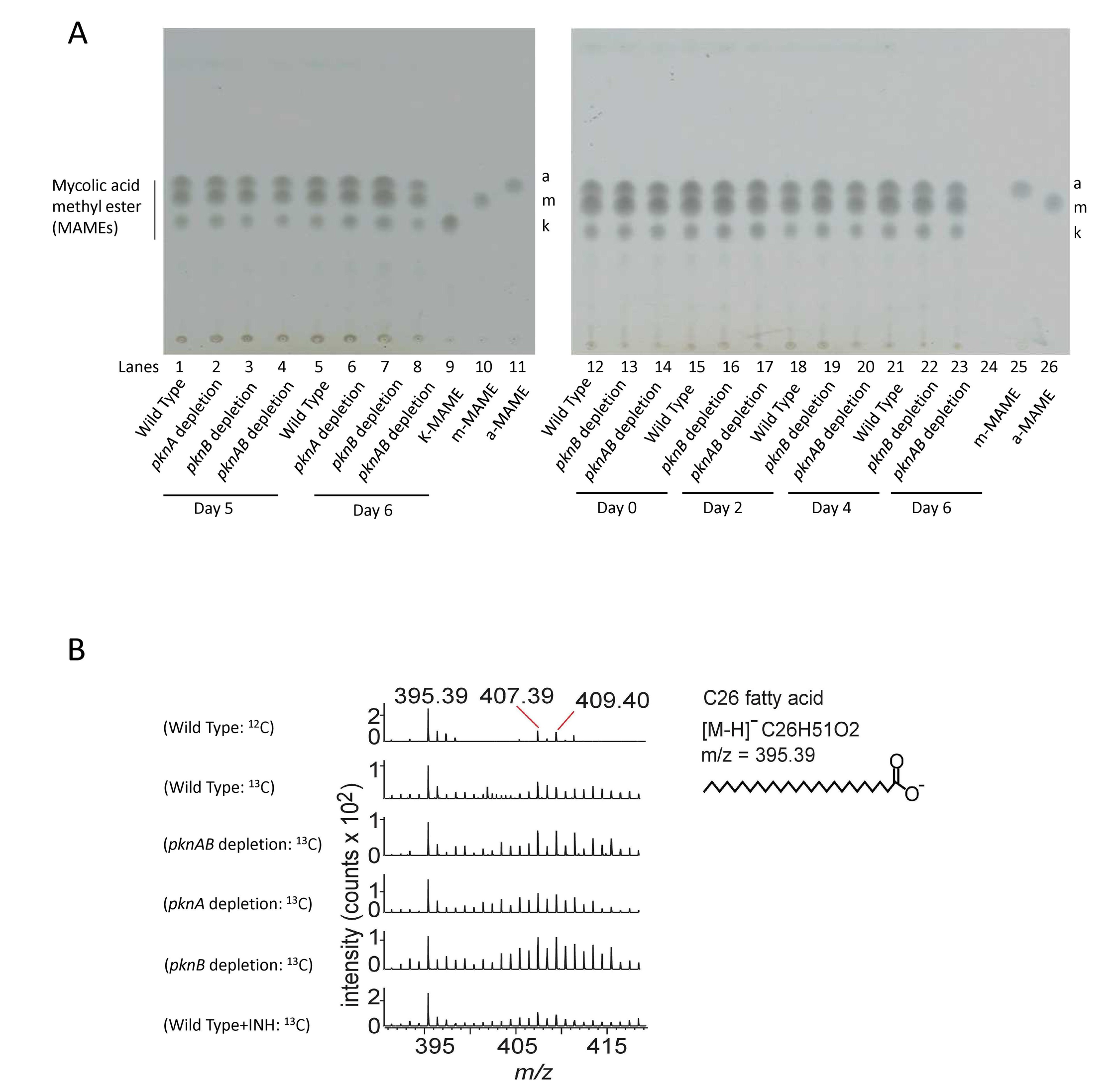

Supplement: S3 Fig — S3A) Thin layer chromatography of saponified cell wall mycolic acids of M. tuberculosis from wild type and kinase depletion strains. M. tuberculosis (wild type), pknA, pknB, pknA+B depletion strains were grown with 0.25 μg/ml pristinamycin at 37°C in 7H9+AND+tw until they reached an OD600 of 0.8. The cells were spun down, washed with PBS-Tx (PBS plus 0.05% tyloxapol), and diluted to an OD600 of 0.1, and then grown +/-0.25 μg/ml pristinamycin in 7H9+AND without tween-80. At each time point, cells were pelleted and washed with PBS. Biological duplicate samples for TLC were harvested at serial time points by resuspending the bacteria into 15 mL 1:2 (V:V) chloroform:methanol to sterilize samples and extract lipids. Total mycolates were isolated by saponification as previously described [32], dried down under nitrogen and analyzed by TLC (Silica Gel 60, Macherey-Nagel) using 3 developments with 90:15 (v/v) hexane: diethyl ether and developed with 8% (v/v) phosphoric acid and 3% (w/v) cupric acetate and charring. S3B) Detection of newly synthesized fatty acids using 13C labeling of wild type and kinase depletion strains. HPLC-MS negative ion mode analysis of the free fatty acid (m/z 395.39) was performed on total lipid extracts from the wild type and kinase depletion strains to measure 13C incorporation from [1,2-13C] acetate uptake. The 13C labeling gave additional peaks from m/z 400 to 415 for all strains. The total lipid extracts used here were also used in experiments shown in Fig 5E and 5F [64]. (TIF) [file ppat.1008452.s007.tif]

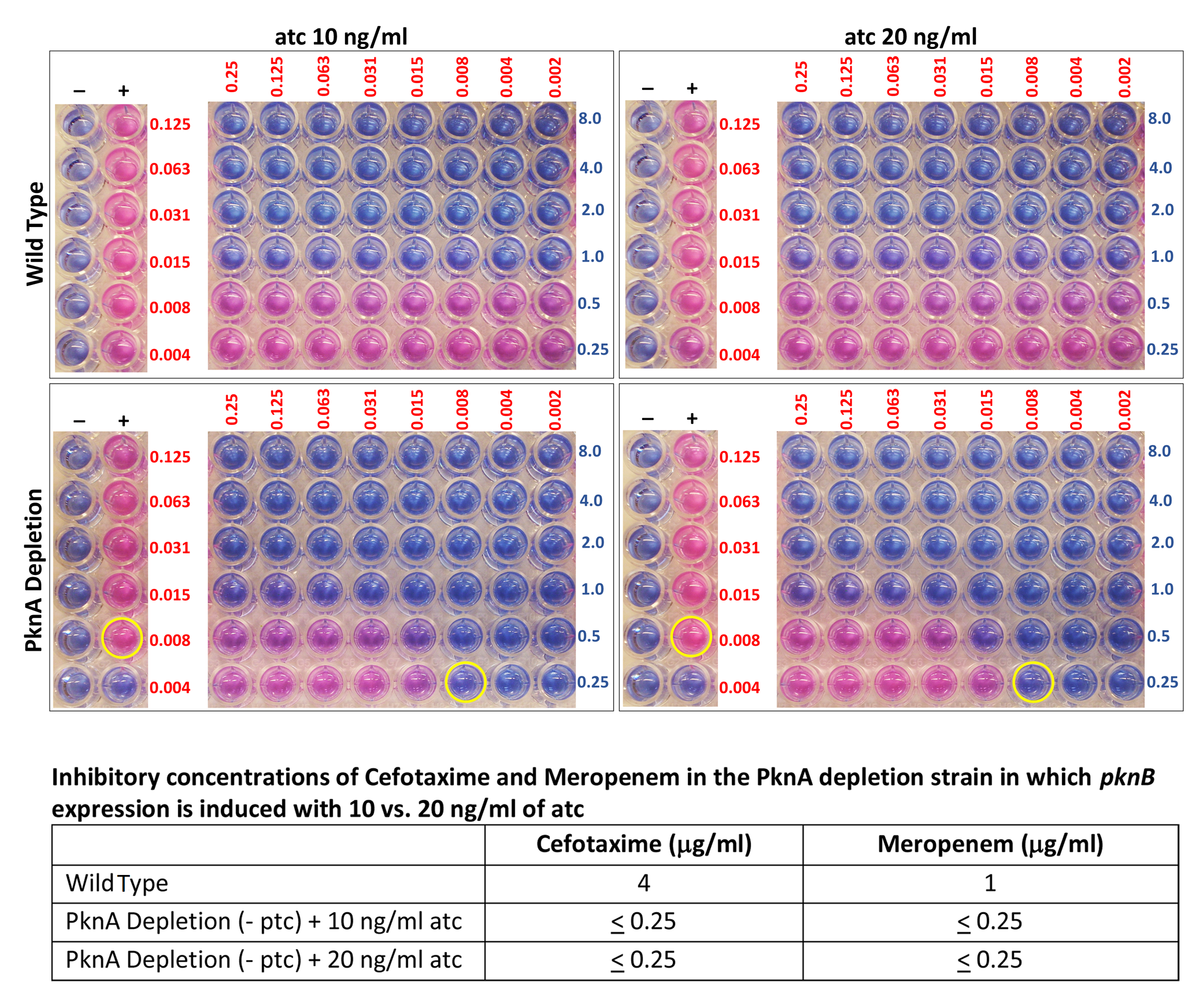

Supplement: S4 Fig — Inhibitory concentrations of cefotaxime and meropenem in wild type and in the pknA depletion strain were determined using the MABA assay as described in the Materials and Methods section. Experiments were performed with pknB expression induced by either 10 or 20 ng/ml of atc. S4A) Representative images of wild type and pknA depletion strains tested for meropenem susceptibility. The - and + signs above the columns at the left side of each image indicate negative control (medium) and positive control (growth in the absence of antibiotic), respectively. The numbers in red indicate the ptc concentration in μg/ml in each row or column. The yellow circle in the positive control column in the lower panels indicates the well with the lowest ptc concentration that allowed growth in the absence of antibiotics (0.008 μg/ml ptc). The yellow circle in the 0.008 μg/ml column on the right side of the lower images indicates the lowest meropenem concentration that inhibited growth at this ptc concentration. S4B) Summary table of results for wild type and pknA depletion strains with cefotaxime and meropenem. The lowest inhibitory concentration in the pknA depletion strain is the same when 10 or 20 ng/ml of atc is used and is shown as ≤ 0.25 μg/ml because this was the lowest concentration of antibiotics included in these experiments. Biological duplicate experiments were performed for all strains. (TIF) [file ppat.1008452.s008.tif]

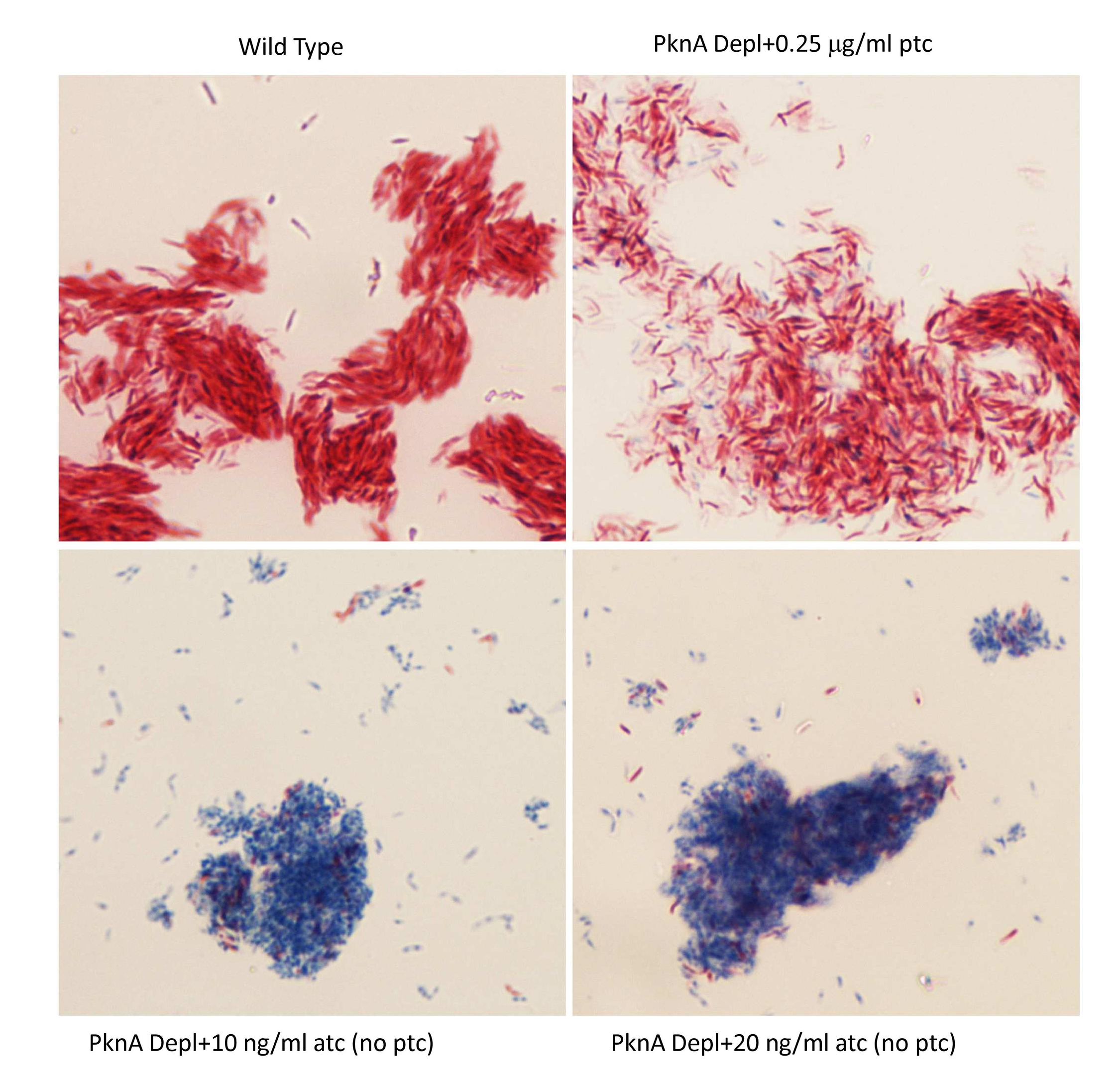

Supplement: S5 Fig — Acid-fast staining was performed as described in the materials and methods section on wild type and pknA depletion strains grown in Middlebrook 7H9 medium. The pknA depletion strain was grown in the presence of ptc to induce expression of both pknA and pknB, or in the presence of 10 ng/ml or 20 ng/ml atc to induce the expression of pknB but not pknA. The wild type and ptc-induced pknA depletion strain show acid fast staining of most cells (bright pink), while most cells the pknA depletion strain grown in the presence of 10 or 20 ng/ml of atc are not acid-fast and stain blue with the counterstain. (TIF) [file ppat.1008452.s009.tif]

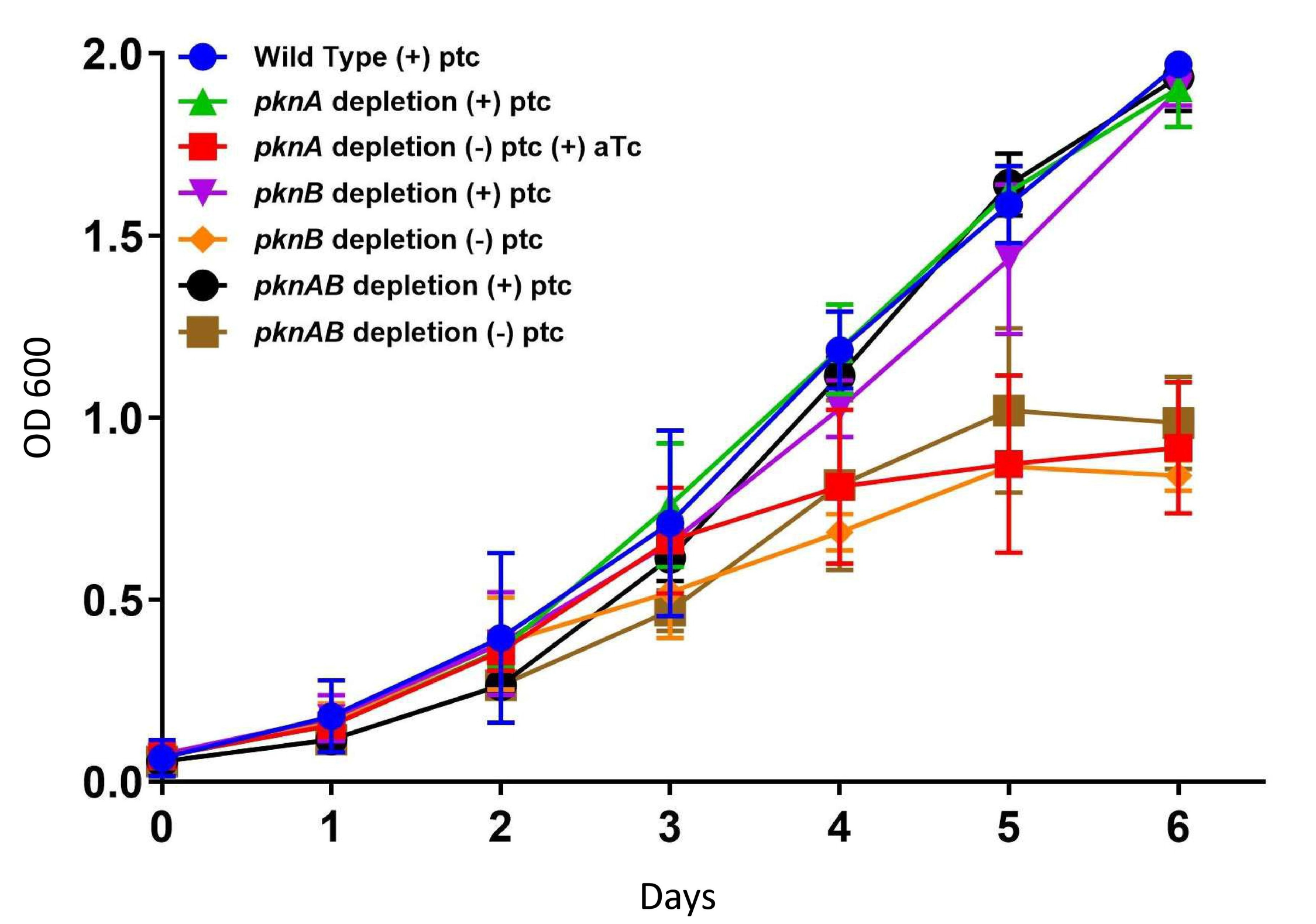

Supplement: S6 Fig — The cells were grown with 0.25 μg/ml pristinamycin until they reached an OD600 of 0.8. The cells were spun down, washed with PBS-Tx (PBS plus 0.05% tyloxapol), and diluted to an OD600 of 0.1, and then grown +/- 0.25 μg/ml pristinamycin in 7H9+AND, plus induction of pknB by 20ng/ml atc for pknA depletion. OD600 measurements were performed every 24h. Note the slower onset of growth arrest for the pknB depletion strain in these cultures with a 10-fold higher inoculum compared to Fig 3. Data are the average of three biological replicates and error bars represent +/- 1 SD. (TIF) [file ppat.1008452.s010.tif]

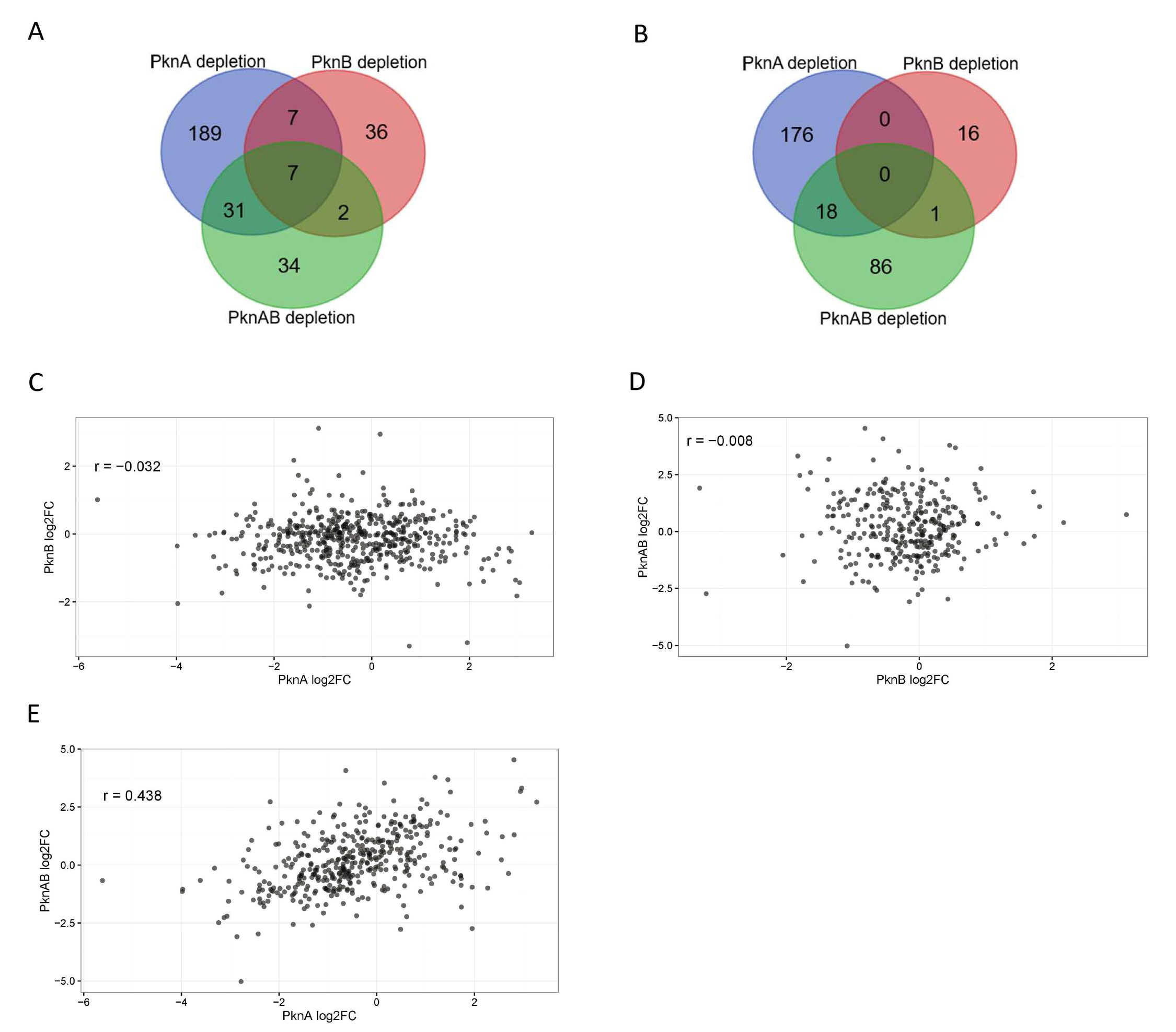

Supplement: S7 Fig — S7A. Venn diagram of the significantly decreased unique phosphopeptides identified in each strain. S7B. Venn diagram of the significantly increased unique phosphopeptides identified in each strain. S7C-E. Pearson correlation analysis of Log2FC of phosphopeptides in kinase-depleted relative to kinase replete bacteria from each of the three depletion strains. Each dot represents a phosphopeptide that was quantified in both of the strains being compared S7C. pknA Log2FC vs. pknB Log2FC, S7D. pknB Log2FC vs. pknAB Log2FC, S7E. pknA Log2FC vs. pknAB Log2FC. (TIF) [file ppat.1008452.s011.tif]

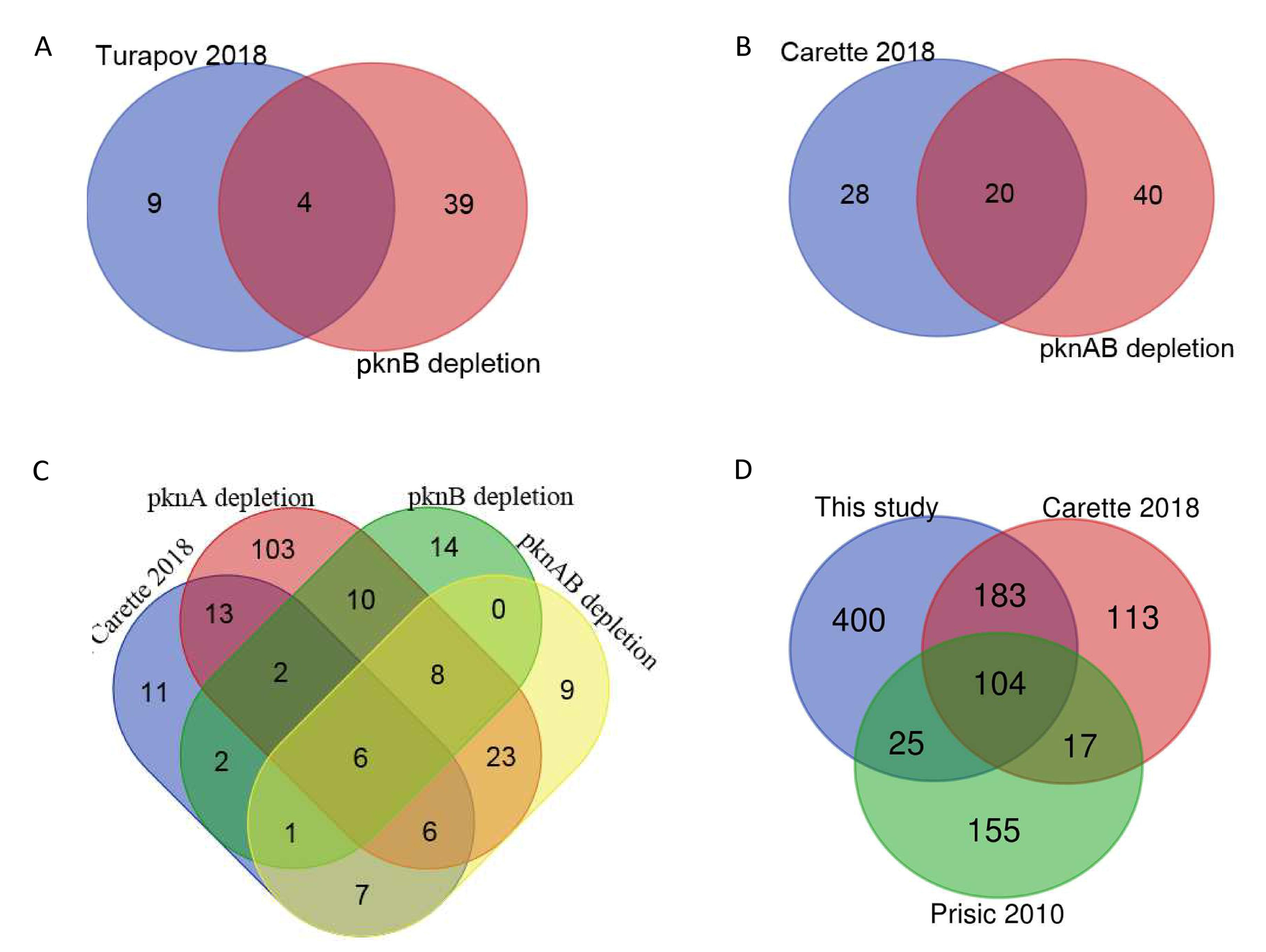

Supplement: S8 Fig — S8A) Overlap of the decreased phosphoproteins identified in pknB depletion data from this study (N = 43, red) compared to phosphoproteins with decreased phosphorylation identified in pknB depletion data from Turapov et al (N = 13, blue) [24]. S8B) Overlap of the decreased phosphoproteins identified in the pknA+pknB depletion data from this study (N = 60, red) compared to decreased phosphoproteins identified in response to treatment with a small molecule chemical inhibitor of both PknA and PknB (Carette, N = 48, blue) [6]. S8C) Overlap of the decreased phosphoproteins identified in this study in pknA depletion (N = 171, red), pknB depletion (green, n = 43), and pknA+pknB depletion (yellow, n = 60) strains compared to proteins showing decreased phosphorylation in response to small molecule inhibition of PknA and PknB in Carette, et al (N = 48, blue). S8D) Overlap of all quantified phosphoproteins in this study (N = 712, blue) compared to two prior phosphoproteomic studies from this laboratory, Carette 2018 (all quantified phosphoproteins, N = 417, red), and a study that identified phosphorylation sites in M. tuberculosis proteins at different growth stages and following exposure to stresses (Prisic 2010, N = 301, green) [6, 7]. (TIF) [file ppat.1008452.s012.tif]

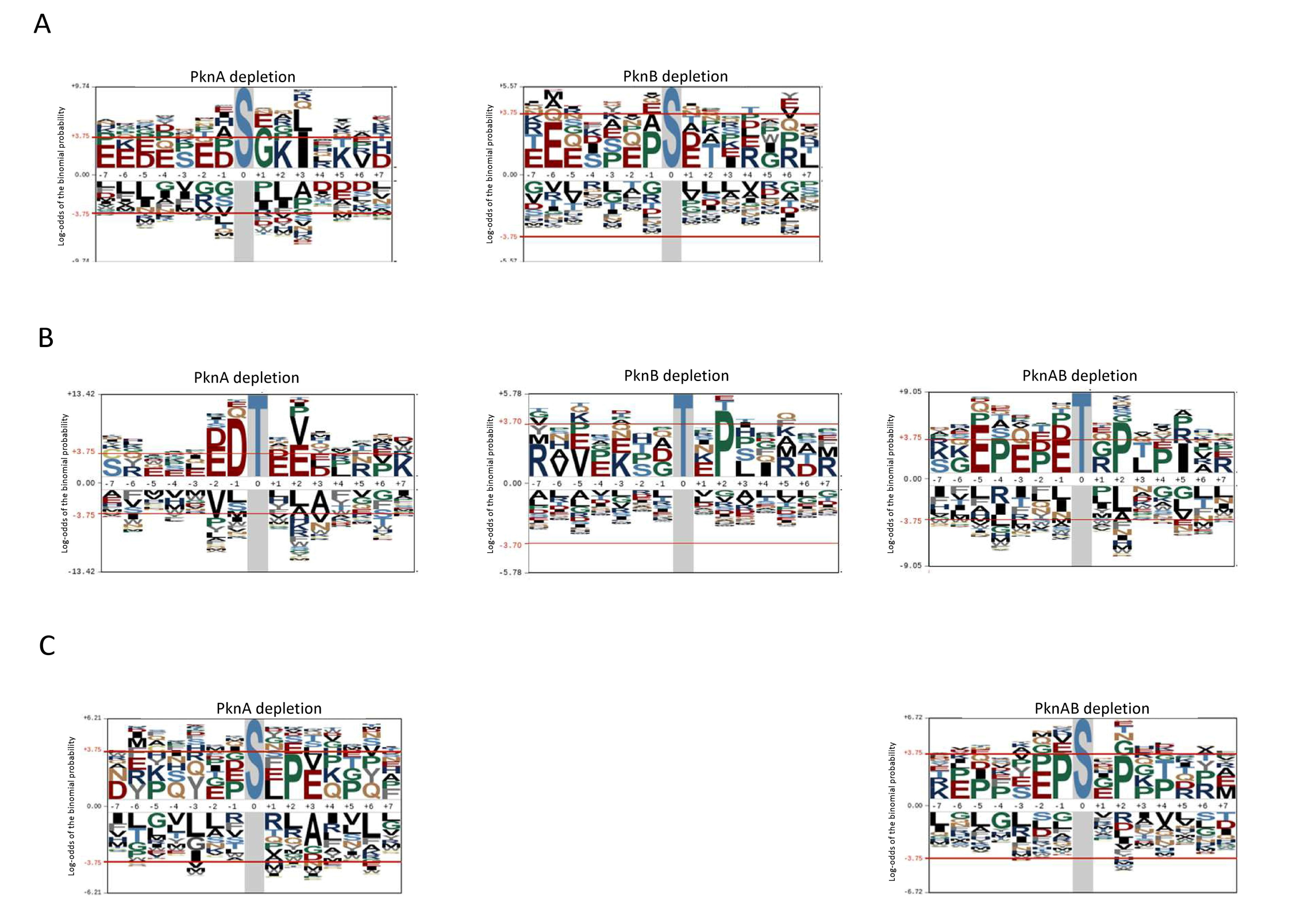

Supplement: S9 Fig — S9A, pLogos showing relative statistical significance of amino acids at positions adjacent to the phosphoacceptor Ser in phosphopeptides that were significantly decreased in the pknA or pknB depletion strains. S9B, pLogos showing relative statistical significance of amino acids at positions adjacent to the phosphoacceptor Thr in phosphopeptides that were significantly increased in each kinase depletion strain. S9C, pLogos showing relative statistical significance of amino acids at positions adjacent to the phosphoacceptor Ser in phosphopeptides that were significantly increased in pknA or pknA+pknB kinase depletion strain (there were too few increased Ser-phosphorylated peptides in the pknB-depletion strain to analyze). The red horizontal line indicates P = 0.05 after Bonferroni correction. (TIF) [file ppat.1008452.s013.tif]
